# Advances in Bioactive Polysaccharide—Small-Molecule Drug Supramolecular Nanocomplexes for Drug Delivery and Therapeutic Applications

**DOI:** 10.3390/jfb17090449

**Published:** 2026-09-06

**Authors:** Mei Zhang, Linjie Zheng, Benyong Lou, Yanjie Zhang, Rongjian Sa, Ling Liang, Li Feng, Longtao Zhang

**Affiliations:** 1 College of Materials and Chemical Engineering, Minjiang University, Fuzhou 350108, China; zhenglinjie831@gmail.com (L.Z.); lby@mju.edu.cn (B.L.); yanjiezhang@mju.edu.cn (Y.Z.); rjsa@mju.edu.cn (R.S.); liangling@mju.edu.cn (L.L.); 17828614532@163.com (L.F.); 2 College of Food Science, Fujian Agriculture and Forestry University, Fuzhou 350002, China

**Keywords:** bioactive polysaccharides, drug delivery, supramolecular nanocomplexes, noncovalent assembly, polysaccharide contribution

## Abstract

Bioactive polysaccharides (e.g., fucoidan, β-glucans, and medicinal plant polysaccharides) contain functional groups that interact with drug molecules, and some also retain their own biological activities. Through reversible noncovalent interactions, they can associate with small-molecule drugs and form supramolecular nanocomplexes, defined here as nanoscale assemblies in which the polysaccharide is a main structural component and its association with the drug contributes to assembly or drug retention. Multicomponent composites and bulk local matrices are discussed separately as related or extended systems. The review covers hydrogen bonding, hydrophobic association, electrostatic complexation, π–π stacking, and the cooperation among these interactions, together with the effects of pH, ionic strength, concentration, and solvent composition. Nanoprecipitation/solvent exchange, polyelectrolyte complexation, direct aqueous self-assembly, and microfluidic-assisted assembly are compared with respect to nanostructure formation, process control, and reproducibility. Molecular, colloidal, solid-state, and computational evidence is examined together when interpreting structure–assembly–performance relationships. Reported advantages include improved drug dispersibility, colloidal stability, release control, bioavailability, cellular uptake, biodistribution, and safety. In some systems, the polysaccharide itself may also contribute to therapeutic effects in tumors, inflammatory diseases, and wound healing. Related local-matrix systems are considered separately. Further development of these nanocomplexes will require better quantitative analysis of assembly mechanisms, more consistent polysaccharide characterization, careful biocompatibility assessment, scalable preparation, and longer-term safety evaluation.

## 1. Introduction

Small-molecule drugs remain indispensable in the treatment of cancer, inflammatory diseases, infections, metabolic disorders, and other complex diseases. However, many potent small-molecule drugs and natural bioactive compounds are limited by poor aqueous solubility, chemical instability, rapid metabolism, short circulation time, insufficient tissue selectivity, and dose-dependent systemic toxicity. As a result, these limitations commonly reduce bioavailability, weaken therapeutic efficacy, and increase the risk of adverse effects. Nanodrug delivery systems (NDDSs) have therefore been developed to address these formulation and delivery problems by improving drug solubilization, protecting labile drugs, regulating release behavior, and promoting drug delivery to diseased tissues [1,2,3]. Among the available carrier materials, natural biological macromolecules are of particular interest. They can serve as structural components in nanoscale formulations and, at the same time, provide biological functions that may affect drug stability, cellular interactions, and therapeutic outcomes.

Polysaccharides are structurally diverse natural macromolecules derived from plants, animals, algae, fungi, and microorganisms. Chitosan, alginate, hyaluronic acid, dextran, cellulose derivatives, fucoidan, β-glucans, and medicinal plant polysaccharides are commonly reported in drug delivery studies [4,5]. Their low toxicity, biodegradability, biocompatibility, renewable origin, and ease of chemical modification make them useful carrier materials [4,5]. These properties, however, do not necessarily mean that the polysaccharide itself is biologically active. Three types of evidence are therefore distinguished here. Intrinsic pharmacological activity refers to effects such as immunomodulatory, anti-inflammatory, antioxidant, antiviral, or antitumor activity demonstrated independently of the loaded drug. Delivery-related biological functions include receptor-associated recognition, mucoadhesion, and interactions with biological barriers. Biocompatibility, biodegradability, and low toxicity are treated as general carrier properties rather than evidence of bioactivity. This distinction is important because biological recognition alone does not prove intrinsic pharmacological activity. For chemically modified polysaccharides, activities reported for the native material are also not assumed to remain unchanged unless the derivative itself has been examined [6,7].

Polysaccharide structure also affects how these materials assemble with small-molecule drugs. Hydroxyl, carboxyl, amino, sulfate, and carbonyl groups influence hydration, charge density, chain conformation, and chemical modification, while also providing sites for hydrogen bonding, electrostatic attraction, hydrophobic association after modification, and carrier–drug interaction [8,9]. Reversible noncovalent association can therefore produce different nanoscale structures. In this review, the term “supramolecular nanocomplex” describes the assembly mechanism rather than a specific morphology. Micelles, nanogels, nanoparticles, nanoaggregates, nanocapsules, host–guest complexes, and polyelectrolyte complexes refer instead to physical architectures or particular modes of assembly. A system is included as a core polysaccharide–drug supramolecular nanocomplex when the polysaccharide is a main structural component, the payload is a small-molecule drug or bioactive compound, a dispersed nanoscale assembly is formed, and reversible polysaccharide–drug association is involved in assembly, drug retention, or both [10,11]. A nanosized polysaccharide particle is therefore not automatically considered a supramolecular nanocomplex simply because it contains a drug and a polysaccharide.

Fungal, medicinal-plant, and animal-derived polysaccharides illustrate the range of materials covered in this review. Their reported functions may arise from intrinsic pharmacological activity, delivery-related biological effects, or both. These roles are considered separately from general carrier properties and, where chemically modified polysaccharides are used, should be supported by evidence obtained from the actual derivative rather than inferred from the native polysaccharide [12,13,14,15,16].

Recent reviews cover parts of this field, but their scope and analytical focus are not the same. Liu et al. [1] reviewed natural polysaccharide-based nanodrug delivery systems for cancer therapy, while Wang et al. [15] summarized traditional Chinese medicine polysaccharides used as nanocarriers. Zeng et al. [17] concentrated on polysaccharide-based nanomedicines for cancer immunotherapy. Zhang et al. [18] took a broader view of self-assembled nanocarriers derived from proteins, polysaccharides, and lipids, organizing these systems around the relationship between assembly, structure, and function. More recently, Zhao et al. [19] reviewed natural small-molecule–polysaccharide nanoparticles with attention to component types, assembly methods, characteristic–efficacy relationships, process variables, and applications beyond drug delivery.

The present review takes a narrower mechanistic view. Its main focus is on nanoscale systems in which the reversible association between a polysaccharide and a small-molecule drug plays a clear role in assembly or drug retention. Particular attention is given to the distinction between the intrinsic pharmacological activity of the polysaccharide and its delivery-related functions. Molecular, colloidal, solid-state, and computational evidence is also considered together, so that assembly behavior can be discussed in relation to formulation performance, reproducibility, safety, and translational limitations. The aim is therefore not to provide a general catalogue of polysaccharide nanocarriers or natural small-molecule–polysaccharide particles, but to examine the evidence linking structure, interaction, assembly, and performance in bioactive polysaccharide–small-molecule drug supramolecular nanocomplexes.

Based on this definition, several types of systems are not included in the core evidence discussed here: (i) purely covalent polysaccharide–drug conjugates without a relevant noncovalent assembly step; (ii) conventional drug-loaded polysaccharide nanoparticles in which the drug is only physically entrapped and direct polysaccharide–drug association has not been demonstrated; (iii) systems in which the polysaccharide mainly acts as a coating or ligand on an inorganic or unrelated polymeric core; (iv) multicomponent herbal or decoction-derived particles and Pickering emulsions in which the contribution of a defined polysaccharide–drug pair cannot be separated; and (v) bulk hydrogels, scaffolds, coatings, and tissue-engineering matrices.

The following sections examine the structure–interaction–assembly–performance relationship within this defined group of nanocomplexes. Section 2, Section 3, Section 4, Section 5 and Section 6 discuss noncovalent interactions and their regulation, preparation methods, mechanistic characterization, delivery performance, biomedical applications, and translational limitations. Systems that fall outside the core definition but remain relevant to the broader field are discussed separately in Section 6.4.

### Literature Search Strategy and Study Selection

A structured literature search was conducted in PubMed, Web of Science Core Collection, and Scopus from database inception to 21 August 2026. The search combined terms related to polysaccharides (e.g., polysaccharide, chitosan, alginate, hyaluronic acid, dextran, fucoidan, β-glucan, and medicinal or fungal polysaccharides) with terms describing small molecules and drug delivery (e.g., small molecule, drug, bioactive compound, drug delivery, encapsulation, release, and bioavailability). Terms related to assembly and interaction mechanisms, including supramolecular, self-assembly, nanocomplex, nanoparticle, micelle, nanogel, nanoaggregate, polyelectrolyte complex, hydrogen bonding, hydrophobic interaction, electrostatic interaction, and π–π stacking, were also included. Boolean combinations were adjusted according to the indexing rules of each database. Only English-language peer-reviewed journal articles and reviews were considered. Reference lists of relevant primary studies and recent reviews were also checked for studies that were not identified by the main keyword searches.

Titles and abstracts were screened first, followed by full-text assessment according to the scope defined above. A primary study was treated as core evidence when four conditions were met: the polysaccharide or its derivative was a main structural component; the payload was a small-molecule drug or bioactive compound; a dispersed nanoscale assembly was formed; and reversible association between the polysaccharide and the small molecule was involved in assembly, drug retention, or both. Studies outside these criteria were not automatically discarded. When they provided useful information on process control, interfacial stabilization, local retention, gastrointestinal persistence, controlled release, or matrix-guided delivery, they were classified as related or extended systems rather than direct mechanistic evidence for the core nanocomplexes. Reviews were mainly used to identify the scope of the field, compare existing viewpoints, and trace relevant references. Mechanistic and quantitative conclusions were based mainly on primary studies.

This article is a critical narrative review rather than a systematic review or meta-analysis. The structured search was therefore used to make the literature selection and scope boundaries clearer, not to claim exhaustive coverage or quantitative pooling. Studies were interpreted according to the relevance and strength of their mechanistic, formulation, and biological evidence within the core and extended categories.

## 2. Formation Mechanism of Supramolecular Nanocomplexes

Polysaccharides and small-molecule drugs can associate through hydrogen bonding, hydrophobic association, electrostatic interactions, π–π-related aromatic association, and other weak forces. These interactions are reversible, and their relative contributions depend on molecular structure and the surrounding medium. A specific interaction should only be assigned when the available evidence is sufficient to support it. Molecular association, nanoscale particle formation, and subsequent formulation or biological effects represent different levels of evidence and should not be interpreted in the same way. Table 1 summarizes representative systems according to this evidence-based approach.

### 2.1. Hydrogen Bonding in Supramolecular Complexes

Hydrogen bonding may contribute to molecular recognition and local stabilization when suitable donor and acceptor groups are available on both components. Hydroxyl, carboxyl, amino, carbonyl, sulfate, and phenolic groups can all take part in such interactions. Their presence alone, however, is not enough to show that hydrogen bonding is the main driving force. More convincing evidence comes from structure-sensitive measurements, comparison of closely related molecules, or experiments that disturb the proposed hydrogen-bonding sites [28].

The chitosan–prenylated flavonoid system reported by Wang et al. [20] provides a useful example. Icaritin (ICT), icariin (ICA), and icariside I (ICS) share similar flavonoid structures but differ in the availability of phenolic hydroxyl groups. Their loading capacities were 16.29%, 5.54%, and 6.58%, respectively. ^^1^H NMR indicated that the 3-OH and 7-OH groups of ICT participated in intermolecular association with amino groups on chitosan. When these hydroxyl groups were substituted by sugar moieties, the interaction became weaker. The difference in loading among the three compounds therefore gives more direct support for a hydrogen-bond contribution than a spectral shift considered alone. Electrostatic interactions were also involved, so the improved acid resistance and salt stability of the nanocomplexes cannot be explained by hydrogen bonding alone.

The curdlan–polyphenol system [21] also supports the involvement of hydrogen bonding together with hydrophobic association, although the individual contribution of each interaction was not separated experimentally. For this reason, the stability and biological results of this system are treated in Table 1 as outcomes of the complete formulation rather than as direct evidence for hydrogen bonding.

### 2.2. Hydrophobic Interactions in Supramolecular Complexes

Hydrophobic association becomes more relevant when a polysaccharide contains hydrophobic regions or is chemically modified to introduce amphiphilic character. Most native polysaccharides are strongly hydrated in water, so fatty-acid, cholesterol, alkyl, or other hydrophobic groups are often introduced to create nonpolar domains. These regions can associate in aqueous media and provide space for poorly soluble small molecules, while the hydrophilic polysaccharide chains remain exposed to water. For this type of assembly, degree of substitution (DS), critical micelle/aggregation concentration (CMC/CAC), carrier/drug ratio, and loading behavior give more useful information than particle formation alone.

SA-BSP micelles [22] and octadecanoate oat β-glucan micelles [23] show this point. In these systems, DS, carrier/drug ratio, and CMC/CAC provide more direct information on hydrophobic self-association than simply observing nanosized particles. Their quantitative values are summarized in Table 1.

Hydrophobic or aromatic modification can also change charge density, hydration, chain conformation, enzymatic accessibility, receptor interactions, biodegradability, and biological activity [10,29]. Properties reported for the native polysaccharide therefore should not be assumed to remain unchanged after modification unless the derivative itself has been evaluated.

### 2.3. Electrostatic Complexation in Supramolecular Complexes

Electrostatic complexation becomes important when the polysaccharide and drug carry complementary charges. Chitosan is positively charged under acidic conditions because of amino-group protonation, whereas alginate, hyaluronic acid, heparin, fucoidan, pectin, and chondroitin sulfate generally contain ionized carboxylate or sulfate groups. The one-pot hyaluronate/doxorubicin (HA/DOX) system provides a useful quantitative example [25]. The HA used in this study had a molecular weight of about 6400 g/mol and contained approximately 16 carboxylate groups per chain. At a [DOX]/[HA] molar ratio of 1, little aggregation was observed. Increasing the ratio to 2 produced very large aggregates (7825 ± 1060 nm; PDI 2.913), whereas a ratio of 5 gave particles of approximately 250 nm. Each milligram of nanoaggregates contained about 0.24 mg DOX, equivalent to approximately 3.5 DOX molecules per HA chain. The authors attributed the assembly to electrostatic ion pairing together with cation–π-related association of DOX. The marked dependence on charge ratio supports the involvement of electrostatic interactions. Turbidity and DLS, however, mainly show the result of assembly and do not indicate how much each interaction contributes.

Zeta-potential reversal, pH-dependent particle formation, salt sensitivity, and changes in ionizable FTIR bands can also indicate electrostatic involvement. None of these measurements alone gives the binding strength, and all can be influenced by buffer composition, dilution, serum proteins, or steric stabilization. A more reliable interpretation therefore needs information on relevant pKa values and charge stoichiometry, together with molecular-level spectroscopy or binding/thermodynamic measurements. Controlled changes in pH or ionic strength are also useful when possible. Salt effects require particular care: screening may weaken attractive interactions, but it can also reduce repulsion between charged chains. Either process can change particle size, swelling, and drug release [30].

### 2.4. π–π Stacking in Supramolecular Complexes

π–π stacking requires aromatic or sufficiently conjugated surfaces on both interacting partners. Most native polysaccharide backbones lack aromatic rings and therefore do not usually interact directly with aromatic drugs through π–π stacking. In polysaccharide-based systems, the aromatic association may instead occur between drug molecules confined within the carrier, or between the drug and an aromatic group introduced during polysaccharide modification. Cation–π association should be considered separately because it follows a different interaction mechanism. Likewise, fatty-acid, cholesterol, and other nonaromatic hydrophobic groups may strengthen hydrophobic association, but they do not provide a direct site for π–π stacking.

The chitosan/DOX molecular-dynamics study [27] illustrates the first case. The protonation state of chitosan affected chitosan–DOX association, whereas π–π stacking occurred mainly between the anthraquinone rings of DOX molecules. In this system, π–π stacking is therefore better described as drug–drug aromatic association within a chitosan-containing environment rather than direct chitosan–DOX π–π binding. Molecular simulation can distinguish these contacts at the atomic level, although the calculated interaction energies depend on the molecular model and protonation state and still need experimental support.

**Figure 1:**
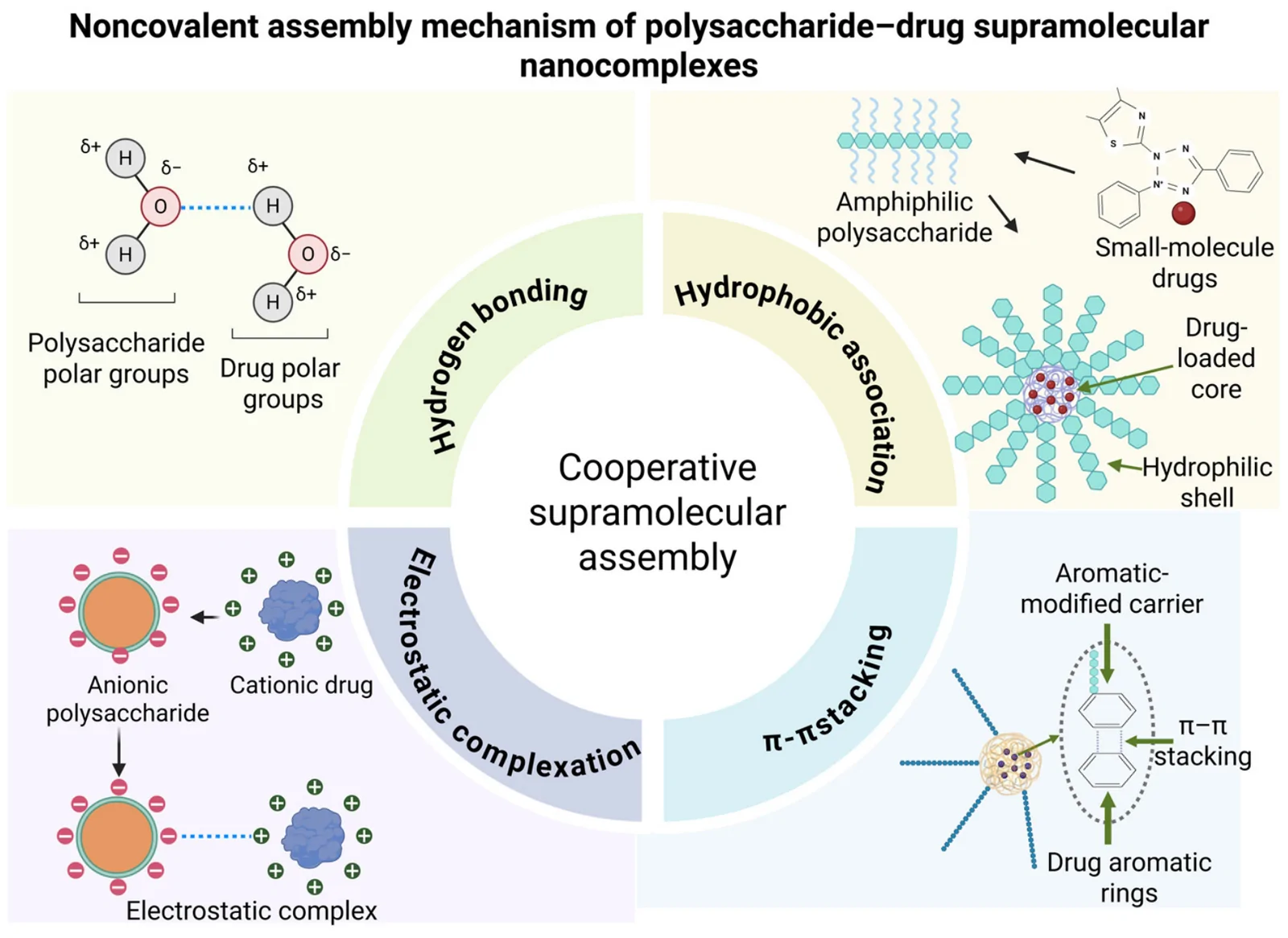
Representative noncovalent interaction types involved in cooperative supramolecular assembly of bioactive polysaccharide–small-molecule drug supramolecular nanocomplexes. The four panels illustrate hydrogen bonding, hydrophobic association, electrostatic complexation, and π–π stacking. Black solid arrows indicate the direction of assembly or complex formation between the depicted components. Blue dashed lines mark the illustrated noncovalent contact between interacting species, including hydrogen-bond-related and electrostatic contacts. Orange arrows indicate panel-specific annotations. The π–π-stacking panel represents carrier–drug stacking between drug aromatic rings and aromatic groups deliberately introduced into the carrier.

A direct carrier–drug π–π interaction becomes more reasonable when the carrier contains a defined aromatic modification. The related CS-g-PZLL co-delivery micelle [26], for example, contains carbobenzyloxy-bearing polylysine segments with aromatic benzyl groups. Fluorescence changes were reported and are consistent with π–π-related association between DOX and the micellar carrier. This evidence is still not specific enough to separate aromatic stacking completely from hydrophobic partitioning. Table 1 therefore indicates whether the aromatic partner is the drug, an introduced aromatic carrier segment, or another component, rather than assigning direct polysaccharide–drug π–π stacking to native polysaccharides. Taken together, these noncovalent interaction modes and their cooperative contribution to supramolecular assembly are summarized schematically in Figure 1.

### 2.5. Cooperative and Hierarchical Assembly Mechanisms

In most bioactive polysaccharide–small-molecule drug supramolecular nanocomplexes, several weak interactions are involved at the same time. Hydrogen bonding may take part in the early recognition of the drug, hydrophobic association may promote formation of a drug-rich core, and electrostatic interactions can contribute to charge compensation and particle nucleation. Aromatic association becomes relevant only in systems containing suitable conjugated groups. The order and relative contribution of these interactions vary with the formulation. Self-assembled hyaluronic-acid/testosterone nanocarriers provide one example: changing the degree of hydrophobic substitution altered the organization of the amphiphilic polysaccharide and its ability to form drug-loaded nanostructures [31].

Parameters such as Ka/Kd, ΔG, ΔH/ΔS, CMC/CAC, degree of substitution, carrier/drug ratio, and simulation-derived interaction energies can help narrow down the possible assembly mechanism. Complete affinity and thermodynamic datasets, however, are still uncommon for core polysaccharide–drug systems. Spectral shifts or particle formation alone should therefore not be used to infer these values. This lack of quantitative interaction data remains a major mechanistic limitation.

The final nanostructure is also affected by assembly kinetics and polysaccharide structure. Solvent-exchange rate, mixing conditions, molecular weight, branching, charge density, and degree of substitution can all influence how the assembly develops. Reporting these variables together with quantitative interaction data would make comparisons among studies more meaningful. Broader issues related to lot-to-lot standardization are discussed in Section 6.5 [4].

### 2.6. Environmental and Formulation Regulation of Cooperative Assembly

Environmental and formulation conditions such as pH, ionic strength, temperature, carrier/drug ratio, total concentration, and solvent composition can change the balance among reversible interactions. Changes in these factors may then affect nanocomplex formation, colloidal stability, and drug release.

pH and ionic strength are especially important for charged and polar interaction networks. pH is a key variable in charged polysaccharide-based assemblies because it changes the ionization state of carboxyl, amino, sulfate, phosphate, and phenolic groups. Once these groups are protonated or deprotonated, electrostatic attraction, hydrogen-bonding patterns, matrix swelling, and pH-responsive release may all be altered [32]. Ionic strength acts through a related but not identical route. A moderate salt level can screen excessive electrostatic repulsion and make the complex structure more compact, whereas high salt concentrations may also screen the attractive forces needed to maintain electrostatically assembled particles. In this case, the absolute zeta potential usually decreases, and the system may show swelling, aggregation, or premature drug release [33]. Thus, pH and ionic strength should be treated as formulation variables that reshape electrostatic complexation and hydrogen-bond rearrangement, not merely as background properties of the medium.

Temperature and carrier/drug ratio can affect molecular mobility, chain rearrangement, nucleation, and aggregation. Excessive heating may weaken hydrogen bonding, disturb hydration, accelerate drug degradation, or promote particle growth. If the amount of carrier is too low, part of the drug may remain unassociated or form unstable aggregates. The carrier/drug ratio is therefore better adjusted according to carrier–drug affinity and the stability of the resulting dispersion rather than treated as a fixed mass ratio [34].

Solvent composition and hydration state also influence drug dispersion, polysaccharide conformation, desolvation, and the strength of noncovalent interactions. Poorly water-soluble drugs may still require a limited amount of co-solvent or a solvent-exchange step. In such cases, solvent polarity and its effect on chain conformation need to be considered together with residual-solvent risk and process reproducibility.

### 2.7. Mechanism-Guided Design of Experiments for Polysaccharide–Drug Nanocomplexes

A practical DoE strategy can also be developed from the interaction framework described above. Relevant material attributes include polysaccharide molecular weight, charge density, branching, and degree of substitution, together with drug properties such as pKa, hydrophobicity, and aromaticity. These attributes can be considered when setting the ranges of pH, ionic strength, polysaccharide/drug ratio, total concentration, solvent fraction, temperature, and mixing conditions in the initial screening design. Where pKa values or CMC/CAC thresholds are known, the experimental range is preferably extended across both sides of these values rather than restricted to a single region.

It is useful to collect responses at more than one level. When experimentally accessible, molecular or thermodynamic information may include Ka/Kd, ΔG, ΔH/ΔS, NMR chemical-shift changes, or interaction energies. Assembly behavior can be followed through CMC/CAC, particle size, PDI, zeta potential, encapsulation efficiency, loading capacity, and the free-drug fraction. Changes in size or drug retention after controlled variation in pH, salt concentration, dilution, or storage can provide an additional measure of formulation stability. Taken together, these responses help distinguish whether better loading or release behavior is associated with altered molecular interactions or mainly with changes in particle organization.

Factors identified during screening can then be examined more closely using a response-surface design, such as a central composite or Box–Behnken design. Optimization is more informative when several responses are considered together rather than when encapsulation efficiency alone is maximized. Depending on the intended application, useful criteria may include acceptable particle size and PDI, sufficient drug association or loading, a low free-drug fraction, stability in the relevant medium, and a response to pH or ionic-strength changes that is consistent with the proposed interaction mechanism. Experiments conducted at the predicted optimum, together with deliberately perturbed conditions, can then be used to test whether the proposed mechanism remains consistent with the observed formulation behavior. In this way, DoE can support both formulation optimization and mechanism testing instead of serving only as an empirical search for the highest loading or encapsulation efficiency.

## 3. Preparation of Polysaccharide–Small-Molecule Supramolecular Nanocomplexes

Preparation conditions need to be adjusted carefully because the interactions responsible for assembly are reversible. Solvent composition, charge state, concentration, and mixing can all affect the final nanostructure. The choice of preparation method is therefore related to both drug solubility and the charge or amphiphilicity of the polysaccharide. Table 2 compares nanoprecipitation/solvent exchange, polyelectrolyte complexation, direct aqueous self-assembly, and microfluidic-assisted assembly in terms of representative formulation metrics, critical process parameters, reproducibility, scalability, and sterilization constraints.

### 3.1. Nanoprecipitation/Solvent Exchange

Nanoprecipitation, also known as solvent displacement or solvent exchange, is commonly used to prepare polysaccharide-based supramolecular nanocomplexes. In this method, solvent–nonsolvent diffusion induces rapid desolvation, after which nucleation and particle growth occur. The final particle properties are affected by several formulation and process variables, including solvent selection, polymer type, drug characteristics, mixing mode, injection rate, and other flow-related parameters [39].

In polysaccharide–small-molecule drug systems, nanoprecipitation is mainly applied to amphiphilic or hydrophobically modified polysaccharides, especially when the loaded drugs have low water solubility. When the solvent is displaced, the hydrophobic parts and the drug-rich regions tend to collect in the formed particles, while the hydrophilic polysaccharide chains remain in the aqueous phase and assist in forming a hydrated stabilizing shell. Particle size, polydispersity, and loading behavior are affected by solvent composition, the organic/aqueous phase ratio, mixing intensity, polymer concentration, and the carrier/drug ratio [40].

Residual organic solvent, local supersaturation, and mixing-sensitive nucleation are the main limitations of nanoprecipitation and may broaden particle-size distributions during scale-up. Method-specific controls are summarized in Table 2, while manufacturing and residual-solvent issues are discussed in Section 6.5.

### 3.2. Polyelectrolyte Complexation

Polyelectrolyte complexation is a mild aqueous method for forming nanocomplexes through electrostatic association among oppositely charged polysaccharides, small-molecule drugs, or counter-polymers. During this process, charged components may associate spontaneously through Coulombic attraction, leading to charge neutralization, chain rearrangement, and nanoparticle formation. This method is particularly suitable for polysaccharides with ionizable groups such as carboxyl, sulfate, phosphate or amino groups, since their charges can be regulated by the pH and ionic conditions of the medium [41].

Compared with nanoprecipitation, polyelectrolyte complexation is more appropriate for certain bioactive polysaccharides, because it is generally carried out in mild aqueous solutions and does not require large amounts of organic solvent. The formation and stability of these complexes are affected by pH, charge ratio, ionic strength, concentration of components, molecular weight and the order of mixing. Among these factors, pH influences the ionization of amino, carboxyl, sulfate and phosphate groups, while the charge ratio affects whether the complexes become compact or remain colloidally stable [42].

Polyelectrolyte complexes are particularly sensitive to pH, ionic strength, charge ratio, and the order in which the components are mixed. Excessive charge neutralization may lead to precipitation, whereas an insufficient charge balance can result in loose particles with low drug-loading efficiency. Additional stabilization may be achieved through secondary weak interactions, surface coatings, or mild cross-linking [43].

### 3.3. Direct Aqueous Self-Assembly

Direct aqueous self-assembly is a simple and environmentally friendly method for forming polysaccharide–drug supramolecular nanocomplexes, particularly when the polysaccharide carrier has inherent amphiphilic character or has been chemically modified to introduce amphiphilicity. In this technique, amphiphilic polysaccharides can associate in water, and the procedure generally requires little or no organic solvent or surfactant, which is suitable for constructing biocompatible drug delivery systems [44].

When the concentration of amphiphilic polysaccharide derivatives exceeds the critical micelle concentration (CMC) or critical aggregation concentration (CAC), hydrophobic segments tend to associate inward and form drug-loading domains, whereas hydrophilic polysaccharide chains remain exposed to the aqueous phase and contribute to colloidal stabilization. Therefore, the hydrophilic–hydrophobic balance, hydrophobic substituent type, molecular weight, and degree of substitution are key structural parameters regulating nanocomplex formation and stability [29].

Histidine/stearic-acid-modified Bletilla striata polysaccharide systems provide an example of direct aqueous self-assembly [45]. Representative formulations produced particles of about 194–215 nm. However, incorporation of the drug still required DMSO followed by dialysis. This shows that self-assembly of the carrier in water does not necessarily mean that a hydrophobic drug can be loaded without the use of a co-solvent.

Direct aqueous self-assembly may also become unstable when the polysaccharide has insufficient amphiphilic character, or when a high degree of modification or excessive concentration promotes aggregation. CMC/CAC, degree of substitution, concentration, and energy input therefore need to be adjusted together.

### 3.4. Microfluidic-Assisted Assembly

Microfluidic-assisted assembly provides a more controllable approach for preparing polysaccharide-based supramolecular nanocomplexes. In microscale channels, fluid behavior can be regulated through laminar-flow diffusion, hydrodynamic focusing, or controlled solvent exchange, which allows mixing to occur in a more reproducible manner. For polysaccharide nanoparticles, microfluidics has been reported to improve process control, formulation reproducibility, and high-throughput screening when compared with conventional bulk preparation [46].

Microfluidic assembly is affected by flow-rate ratio, total flow, channel geometry, concentration, solvent composition, and drug/carrier ratio. These factors influence mixing, local supersaturation, nucleation, and subsequent particle growth. Its main advantage therefore lies in improved control over the assembly process rather than in introducing a different noncovalent mechanism.

**Figure 2:**
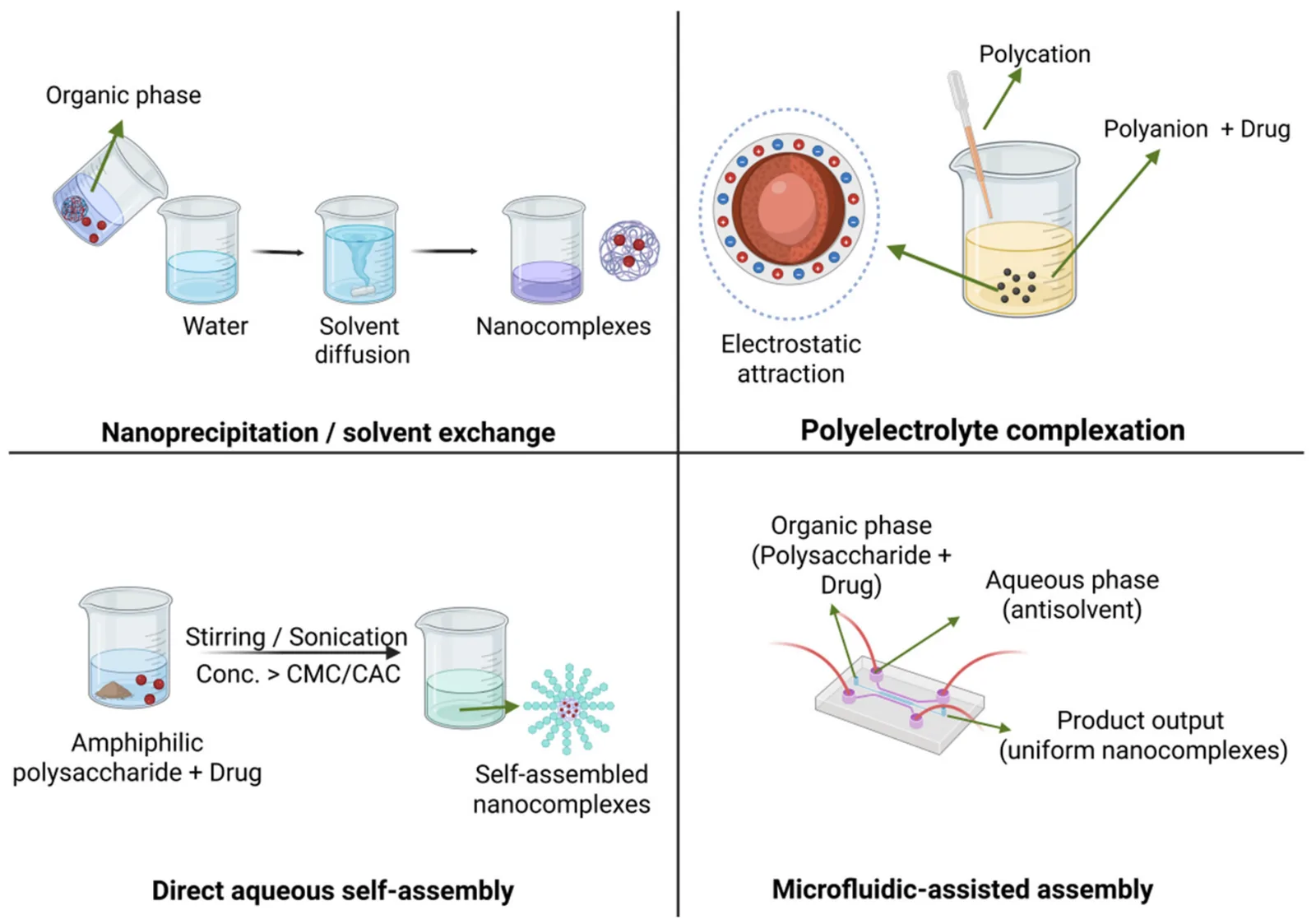
Representative preparation routes for bioactive polysaccharide–small-molecule drug supramolecular nanocomplexes. The four panels summarize nanoprecipitation/solvent exchange, polyelectrolyte complexation, direct aqueous self-assembly, and microfluidic-assisted assembly. Black solid arrows indicate the sequential preparation step or progression from the starting components toward nanocomplex formation. The blue dashed arrow in the polyelectrolyte-complexation panel highlights the charge-mediated noncovalent association underlying complex formation and is not a process-flow arrow. CMC, critical micelle concentration; CAC, critical aggregation concentration.

Practical limitations include high viscosity, back-pressure, clogging, solvent/device compatibility, and the need for process monitoring [46,47,48]. Practical limitations include high viscosity, back-pressure, clogging, solvent/device compatibility, and the need for process monitoring [46,47,48]. Table 2 compares method selection and reproducibility. For a more direct comparison of the preparation processes discussed above, the four representative routes are illustrated schematically in Figure 2.

## 4. Structural Characterization and Mechanistic Evidence Chain

Structural characterization is more useful when each method is linked to a specific question, such as molecular association, colloidal or morphological state, drug physical state, or consistency with computational analysis. No single technique can establish the complete assembly mechanism.

Accordingly, the characterization evidence in Section 4 is discussed at three levels: molecular interactions; morphology, colloidal properties, and solid-state behavior; and computational analysis. Heterogeneity of the polysaccharide is considered where it directly affects interpretation of the measurements.

Table 3 compares routine and advanced characterization methods in terms of the type of evidence they provide, the information obtained, their advantages, and their limitations, with particular consideration of heterogeneous natural polysaccharides.

### 4.1. Molecular Interaction Evidence

Molecular interaction methods are most useful when they can distinguish true association from simple co-dispersion and, where possible, provide information on interaction strength or dynamics rather than relying on a single spectral change.

FTIR and conventional NMR can reveal changes in functional groups or the local molecular environment, although overlapping polysaccharide bands and hydration often make assignment difficult [59,60]. DOSY-NMR adds translational-diffusion information and may help distinguish free from associated species, but signal overlap and the need for sufficient sample concentration remain practical limitations [61].

UV–vis and fluorescence measurements can reflect changes in the microenvironment of conjugated or fluorescent drugs. However, quenching and spectral shifts may also result from concentration effects, inner-filter effects, scattering, photobleaching, or baseline distortion. These observations are therefore more convincing when supported by structure-sensitive or quantitative measurements rather than interpreted alone.

When enough material is available and the binding behavior can be described with a suitable model, isothermal titration calorimetry (ITC) can provide binding stoichiometry, affinity, and thermodynamic parameters such as ΔH, ΔG, and ΔS. This makes it useful for testing interaction hypotheses that cannot be quantified from FTIR or fluorescence alone [62]. Surface plasmon resonance (SPR) provides label-free, real-time information on association, dissociation, and affinity, but immobilization of one binding partner can introduce effects related to surface presentation, mass transfer, and heterogeneous binding sites [63]. Natural polysaccharides with broad molecular-weight distributions may show non-single-site behavior in both ITC and SPR measurements. In such cases, the fitted binding model and the characteristics of the polysaccharide batch need to be reported clearly.

### 4.2. Morphology, Colloidal, and Solid-State Evidence

Morphological, colloidal, internal-structure, and solid-state methods provide different types of information and are not interchangeable. DLS and zeta potential, for example, mainly describe the dispersed state of the particles rather than molecular binding.

TEM shows particle morphology and gives an approximate dry-state size, whereas cryo-TEM is better suited to preserving hydrated soft structures [64]. DLS estimates hydrodynamic size from Brownian diffusion and is particularly sensitive to larger particles in polydisperse samples. It is therefore more appropriately used to describe colloidal size and size distribution than to support molecular association.

For DLS measurements to be reproducible, several experimental details need to be reported. These include temperature; dispersant composition, pH, ionic strength, and viscosity; sample concentration and dilution; equilibration conditions; instrument and detection settings, including scattering angle or detection geometry; independent batches or replicates; and whether the reported distributions are intensity-, volume-, or number-based [65]. The z-average diameter and PDI are better reported separately from transformed size distributions.

Zeta potential is calculated from electrophoretic mobility and can vary with medium composition, pH, ionic strength or conductivity, temperature, viscosity, concentration, dilution history, and the conversion model used. Changes in zeta potential can indicate differences in surface-charge organization, but they do not directly demonstrate polysaccharide–drug binding. A fixed |ζ| value also cannot be applied universally as a criterion for colloidal stability [66]. Stability is better evaluated from time-dependent changes in size and PDI, aggregation behavior, redispersibility, and measurements performed in the relevant medium.

XRD, DSC, and TGA provide information on detectable drug crystallinity and thermal behavior. The disappearance or weakening of diffraction peaks or thermal transitions, however, may also result from low drug loading, matrix dilution, peak overlap, or an amorphous background. Such changes alone do not prove complete molecular dispersion. SAXS and SANS add solution-state information on particle dimensions and internal organization, although interpretation can become model-dependent in heterogeneous systems [67].

Heterogeneity of the carrier also needs to be considered before differences in assembly are attributed to a particular mechanism. SEC-MALS and analytical ultracentrifugation can provide information on molar-mass or sedimentation heterogeneity, while NTA complements DLS by providing number-based particle sizing and particle concentration. These methods are still influenced by their own separation, dilution, and detection conditions [67,68].

### 4.3. Computational Evidence and Integrated Interpretation

Molecular docking and molecular dynamics (MD) can provide atomistic information on possible binding sites, orientations, interaction patterns, and conformational stability. They are useful for examining hydrogen bonding, electrostatic interactions, hydrophobic association, van der Waals forces, and π–π-related contacts. However, these calculations remain supporting evidence and cannot by themselves demonstrate that a nanocomplex has formed.

Docking is mainly used to identify plausible binding regions and interaction modes, while MD follows the behavior of a proposed complex over time in a solvated environment. Parameters such as RMSD, RMSF, radius of gyration, solvent-accessible surface area, hydrogen-bond persistence, and interaction or binding energies can help evaluate structural stability and changes in molecular contacts. Chitosan–curcumin simulations provide one example [69].

The reliability of these calculations is affected by the carbohydrate model, force field, ionization state, solvent, counterions, substitution pattern, and simulation time [70,71]. This is particularly important for heterogeneous natural polysaccharides, whose structural complexity is difficult to reproduce fully in simplified computational models. Docking and MD are therefore more suitable for testing possible interaction pathways and stability trends than for replacing experimental characterization.

## 5. Performance Enhancement and Structure–Assembly–Performance Relationships of Supramolecular Nanocomplexes

Section 5 discusses drug incorporation, apparent solubilization, colloidal, chemical, and physiological stability, release, transport, and safety as separate measurable outcomes. Free, precipitated, surface-adsorbed, and nanocomplex-associated drug represent different physical states and should not be interpreted in the same way.

Encapsulation or entrapment efficiency (EE) refers to the fraction of the initial drug that remains associated with the nanocomplex after the non-associated fraction has been separated using a validated method: EE (%) = associated drug mass/initial drug mass × 100. Drug loading (DL) should state its denominator explicitly; when expressed relative to recovered drug-loaded nanocomplex mass, DL (%) = associated drug mass/recovered nanocomplex mass × 100. If drug content is normalized only to carrier mass, it is more appropriately reported as a drug-to-carrier ratio. Association efficiency and total drug content describe different operational measurements and should not be considered equivalent to EE or DL. They also do not by themselves demonstrate that the drug is physically encapsulated within the nanocomplex. Separation procedures can introduce additional error through particle loss, nonspecific adsorption, dilution-induced drug release, incomplete separation, or co-isolation of precipitated drug. Reporting recovery or mass balance together with appropriate controls can therefore make these measurements more reliable [72].

### 5.1. Apparent Solubility, Supersaturation, and Drug Physical State

Poor aqueous solubility and unstable crystalline states limit the use of many hydrophobic small-molecule drugs and natural bioactive compounds. In polysaccharide–drug supramolecular nanocomplexes, the observed improvement often reflects higher apparent solubility, kinetic supersaturation, faster dissolution, or better aqueous dispersibility rather than a true increase in equilibrium molecular solubility. Apparent solubility may include truly dissolved drug together with nanoscale colloid-associated drug and, when separation is incomplete, very small precipitated particles. Molecular or thermodynamic solubility is therefore better reserved for measurements obtained under defined equilibrium conditions using a validated procedure that excludes colloidal and precipitated fractions. Hydrophobic-domain encapsulation, hydrogen-bond or electrostatic association, and suppression of crystallization can increase the amount of drug dispersed in water without necessarily changing its intrinsic equilibrium solubility [73,74].

The amorphous curcumin–chitosan nanoplex provides a useful quantitative example. At pH 4.4 and a chitosan-to-curcumin charge ratio of 0.8, particles of approximately 260 nm were obtained with >90% complexation efficiency and >80% drug payload. In the presence of hydroxypropyl methylcellulose, dissolution produced a curcumin concentration about five times higher than its crystalline saturation solubility and maintained this supersaturated state for approximately 3 h [75]. This finding is better described as prolonged supersaturation or improved apparent solubility rather than a fivefold increase in molecular solubility. A later scale-up and storage study showed that the same formulation concept maintained its practical solubility-enhancement performance in the dry state, while crystallization-inhibiting excipients helped preserve the amorphous form during long-term storage [76].

For solubility measurements, the medium, temperature, time, separation procedure, analytical recovery, and nature of the reported endpoint need to be stated clearly. In particular, it should be clear whether the value represents equilibrium solubility, kinetic supersaturation, or colloid-associated drug. Similarly, disappearance or weakening of drug signals in XRD or DSC supports reduced detectable crystallinity but does not by itself prove complete amorphization or molecular solubilization. A more convincing interpretation comes from combining solid-state characterization with dissolution or supersaturation measurements and evidence of recrystallization behavior during storage.

### 5.2. Colloidal, Chemical, and Physiological Stability

Colloidal, chemical, and physiological stability describe different aspects of formulation behavior and are best evaluated separately under defined conditions and over a defined period. Colloidal stability concerns whether hydrodynamic size, PDI, dispersion state, and redispersibility are maintained during storage or after challenges such as changes in pH, salt concentration, dilution, or serum exposure. Zeta potential can help explain these changes but is not sufficient on its own to establish stability. Chemical stability refers to processes such as drug degradation, leakage, oxidation, hydrolysis, photolysis, or crystallization. Physiological stability describes how well the drug-containing assembly is maintained, or how it changes, in simulated gastrointestinal fluids, serum, mucus, or other biologically relevant media [77,78,79].

Stabilization by the polysaccharide may result from hydration, steric shielding, surface charge, or confinement of drug-rich regions. Evidence for a more direct contribution from polysaccharide–drug association is stronger when the drug-containing nanoscale assembly can be shown to remain intact under the tested conditions.

In amorphous curcumin–chitosan nanoplexes, increasing the molecular weight of chitosan increased particle size but also improved colloidal stability and prolonged supersaturation, while several other formulation properties changed little [80]. This result shows that stability cannot be judged reliably from a single particle-size or zeta-potential measurement. Longer-term storage studies are still needed to determine whether these properties are maintained over time [76].

For stability studies, experimental conditions such as storage duration, temperature, concentration, medium, pH, ionic strength, dilution history, and any drying or redispersion step need to be reported clearly. The measurements can then be chosen according to the type of stability being examined, for example longitudinal size and PDI, aggregation, drug retention, chemical assay, or solid-state characterization.

### 5.3. Release Methodology and Controlled/Stimuli-Responsive Release

Release studies are easier to compare when the formulation dose, medium composition and volume, pH, ionic strength, temperature, agitation, surfactant or protein content, sink conditions, sampling procedure, replacement volume, and cumulative-release correction are reported clearly. A free-drug control is particularly important, while a physical mixture can be useful when it helps distinguish true assembly effects from simple coexistence of the components. For membrane-based methods, additional details such as membrane material and MWCO, donor and receiver volumes, and effective diffusion area are also needed.

Dialysis introduces an additional membrane-transport step between drug release from the carrier and its appearance in the receiver phase. Slow membrane permeation, membrane adsorption, or insufficient sink conditions may therefore produce an apparently sustained profile [81]. The free-drug control can be used to check whether diffusion across the membrane is faster than release from the carrier. Drug solubility in the actual release medium is also needed to judge sink conditions, while serial sampling requires appropriate correction. Membrane recovery or adsorption and, where feasible, an endpoint donor–membrane–receiver mass balance can provide further checks on drug recovery. If membrane transport becomes rate-limiting, the resulting curve is better described as an apparent dialysis release profile rather than direct carrier-release kinetics.

Release models are useful for comparing formulations and describing release behavior, but they do not by themselves establish a unique physical mechanism. Mechanistic interpretation becomes more reliable only when membrane transport, sink conditions, and the assumptions of the fitted model have also been examined.

### 5.4. Bioavailability, Cellular Uptake, and Biodistribution

Improved bioavailability reflects several structure- and formulation-dependent processes and cannot be inferred from particle formation or apparent solubility alone. The most relevant endpoints depend on the route of administration and may include permeability, cellular uptake, pharmacokinetic parameters such as Cmax, Tmax, AUC, and half-life, and absolute drug concentrations in tissues. Increased cellular uptake does not by itself identify the uptake pathway. A specific internalization mechanism is more convincing when supported by inhibitor studies, receptor blocking, comparisons between receptor-positive and receptor-negative models, or complementary imaging approaches.

Bioavailability and biodistribution are also better interpreted as route-specific quantitative outcomes. For oral delivery, permeability and systemic exposure are usually more informative, whereas parenteral systems require consideration of serum stability, pharmacokinetics, tissue or lesion distribution, and clearance. Fluorescence imaging can show spatial distribution, but quantitative drug analysis is still needed when the fluorescent label may dissociate from the carrier or the drug.

### 5.5. Safety, Impurities, and Toxicity Reduction

Safety needs to be considered separately for the source polysaccharide, any chemically modified derivative, and the final drug-loaded nanocomplex. Source polysaccharides may contain residual proteins, nucleic acids, endotoxin or pyrogens, salts, low-molecular-weight contaminants, and other source-dependent impurities. Chitosan is one example in which residual protein and endotoxin levels require particular attention [82]. Chemical modification introduces an additional impurity profile, including possible residual reagents, catalysts, crosslinkers, organic solvents, unreacted substituents, and degradation products. The final formulation adds further variables such as the drug itself, excipients, free-drug fraction, process-related contaminants, particle size and surface properties, and route-specific exposure. For this reason, the known biocompatibility of the starting polysaccharide cannot be taken as evidence that the modified derivative or the final nanocomplex is also safe.

Endotoxin control becomes especially important when inflammatory or immunomodulatory responses are used to support the biological activity of a polysaccharide. Nanomaterials may adsorb endotoxin and can also interfere with endotoxin assays. Cytokine production or macrophage activation can therefore be wrongly attributed to the carrier if contamination and assay interference are not excluded using appropriate controls [83]. For parenteral nanocomplexes, normal-cell viability alone provides only limited safety information. Additional evaluation may include hemolysis, complement activation, cytokine and inflammatory responses, coagulation or thrombogenicity where relevant, serum and protein-corona interactions, immunogenicity, hematology and clinical chemistry, organ accumulation, histopathology, and repeated-dose toxicity [84]. Local tolerance also needs to be considered according to the intended route, including oral, pulmonary, ocular, dermal, and injectable administration.

## 6. Biomedical Applications and Evidence for Polysaccharide Contribution

The performance of a bioactive polysaccharide–small-molecule drug supramolecular nanocomplex may result from improved drug delivery, delivery-related biological functions of the polysaccharide, intrinsic pharmacological activity of the polysaccharide, or a combination of these effects. Better performance than the free drug alone does not necessarily indicate synergy. Here, synergy is considered only when appropriate controls for the individual components are included and the combined effect exceeds the expected additive response. When both the polysaccharide and the small molecule can be tested as active components, dose–response matrices together with models such as Bliss independence, Loewe additivity, highest-single-agent (HSA), zero-interaction-potency (ZIP), or a justified combination-index method can be used to evaluate the interaction. Because different models may lead to different conclusions, the model and analysis procedure need to be reported clearly [85]. When such evidence is not available, terms such as “enhanced formulation performance,” “polysaccharide contribution,” or “cooperative carrier–drug effect” are more appropriate than “synergy.”

Section 6.1, Section 6.2 and Section 6.3 discuss representative applications within the core scope, while Table 4 provides a quantitative comparison across studies. Related systems that fall outside the core definition are discussed separately in Section 6.4.

### 6.1. Tumor Therapy: Receptor-Associated Uptake, Drug Resistance Modulation, and Carrier Contributions

Receptor-associated uptake can contribute to tumor-cell internalization, but the strength of the targeting claim needs to match the available evidence. A correlation between receptor expression and increased uptake is more appropriately described as receptor-associated uptake. Evidence becomes stronger when competitive inhibition, receptor blocking, receptor knockdown, or comparisons between receptor-positive and receptor-negative cells are included. Active targeting requires more than increased uptake alone. Where feasible, comparison with a matched untargeted formulation, together with receptor perturbation and in vivo evidence showing altered tissue or cellular localization, provides a more convincing basis for this claim. Increased bulk tumor accumulation is not sufficient by itself because vascular permeability, circulation time, particle size, and nonspecific retention can produce a similar result [92]. Self-assembled hyaluronic acid/doxorubicin nanoaggregates illustrate this limitation. HA–CD44 interactions can explain receptor-associated uptake, but enhanced uptake without receptor-blocking experiments is not enough to establish active targeting [25,93].

Tumor-oriented nanocomplexes may also influence intracellular drug disposition and multidrug resistance (MDR) through changes in drug-efflux transporters, intracellular trafficking, lysosomal sequestration, detoxification pathways, or apoptosis. Evidence for these mechanisms is more convincing when intracellular drug retention, transporter activity or expression, apoptosis-related responses, and in vivo treatment outcomes are examined together rather than inferred from drug loading or cytotoxicity alone.

### 6.2. Inflammatory Disease Intervention: Lesion-Preferential Delivery, Macrophage-Associated Interaction, and Microenvironment-Responsive Release

Inflammatory tissues often show barrier disruption, immune-cell activation, oxidative stress, enzyme overexpression, and increased permeability. Nanocomplexes may improve local retention, release behavior, or interactions of anti-inflammatory small molecules with immune cells. Some polysaccharides may also contribute independent immunoregulatory or barrier-related effects [94]. These contributions need to be considered separately from the delivery effect of the nanocomplex.

Several mechanisms may contribute to inflammatory intervention, including preferential permeation into diseased tissue, immune-cell-associated or receptor-mediated interactions, and release triggered by the local microenvironment. Barrier disruption in inflamed skin, intestinal mucosa, or synovial tissue can increase local nanoparticle penetration or retention, but this is better described as lesion-preferential delivery rather than active targeting. Evidence for receptor-mediated uptake becomes more convincing when the proposed pathway is directly perturbed. The peptide-decorated hyaluronic-acid nanovesicle reported by Hu et al. [95] provides a representative non-tumor example. In this system, grafted FmocFF domains formed vesicular structures, and curcumin was incorporated through π–π interactions. The formulation also improved inflammation-related outcomes in rheumatoid-arthritis models. These effects arise from several features of the system, including lubrication, carrier architecture, and curcumin delivery. Without a quantitative additivity analysis, they are therefore better described as a cooperative multifunctional effect rather than established pharmacological synergy.

The evidence base for inflammatory applications is still smaller than that for tumor-related systems. Disease-site exposure, local uptake or retention, inflammatory biomarkers, biodistribution, and safety therefore need to be interpreted within the limits of the experimental model. Expanding this section with mechanistically unrelated systems would not necessarily strengthen the evidence.

### 6.3. Wound Healing: Local Drug Exposure, Infection Control, and Tissue Repair

Wound healing is a local therapeutic setting in which poor drug solubility or limited residence time may occur together with inflammation, impaired tissue repair, and, in infected wounds, microbial burden. For these systems, the wound model needs to be defined clearly, and improved local drug exposure should be distinguished from matrix-related or biological effects contributed by the polysaccharide.

The self-assembling Bletilla striata polysaccharide–azithromycin system provides a representative example [96]. The nanoscale BSP/azithromycin precursor fits the core discussion of noncovalent assembly, whereas incorporation of these nanomicelles into a Carbomer/gelatin hydrogel produces a local matrix and is therefore better considered an extended formulation. The therapeutic performance of the final hydrogel cannot be attributed directly to the dispersed binary nanocomplex alone. In the quantitative comparison in Table 4, an independent oligochitosan–curcumin nanoplex provides a core wound-healing example and shows faster wound closure than native curcumin. However, because a complete set of polysaccharide-alone and physical-mixture controls was not included, the result supports improved local drug delivery rather than formally demonstrated carrier–drug synergy.

For core wound-healing systems, local drug exposure and wound-healing outcomes are most informative when controls allow the contributions of the small molecule and the carrier to be separated. Fewer studies currently meet these strict core criteria than in tumor delivery, while regenerative scaffolds are more often classified as extended systems. This limitation reflects the present evidence base rather than a need to broaden the core category with mechanistically different formulations.

### 6.4. Related and Extended Polysaccharide-Based Delivery Systems Outside the Core Scope

Related emulsions, multicomponent or inorganic particles, hydrogels, and regenerative scaffolds fall outside the core definition of a dispersed polysaccharide–small-molecule supramolecular nanocomplex and are discussed separately. They are included here only when they provide useful information on interfacial stabilization, process control, local retention, controlled release, or matrix-supported tissue repair.

**Figure 3:**
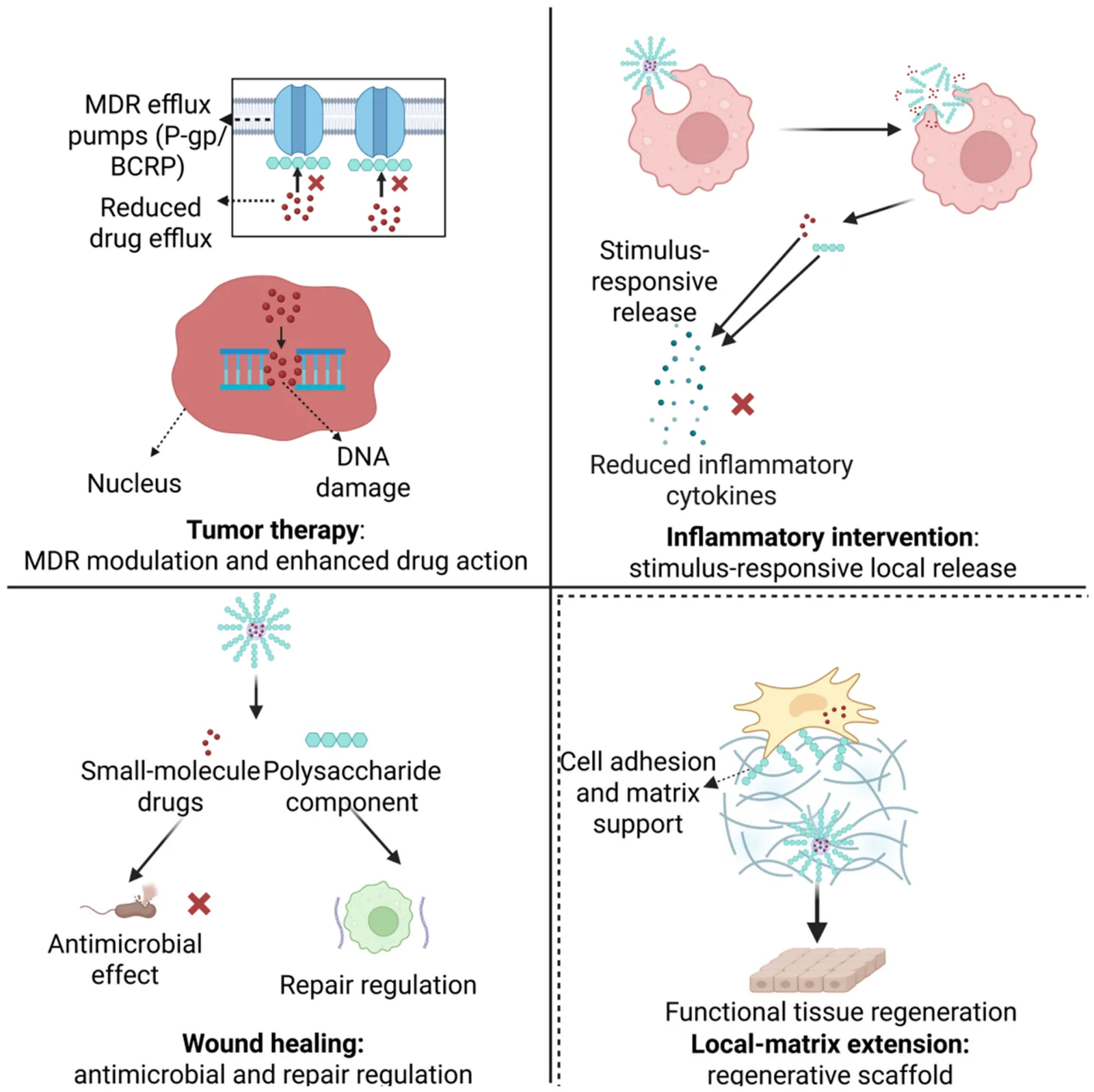
Representative biomedical application pathways and related local-matrix extensions of bioactive polysaccharide–small-molecule drug supramolecular nanocomplexes. The (**upper panels**) illustrate tumor therapy through multidrug-resistance modulation and inflammatory intervention through stimulus-responsive local release, whereas the (**lower panels**) illustrate wound healing through antimicrobial and repair-related effects and a related local-matrix/regenerative-scaffold extension. Black solid arrows indicate the direction of the depicted delivery, biological, or downstream outcome pathway. Black dotted arrows are annotation/leader lines linking labels to the illustrated structures or endpoints (e.g., MDR efflux pumps, reduced drug efflux, nucleus, and DNA damage) and should not be interpreted as separate mechanistic pathways. The black dashed border identifies the local-matrix/regenerative-scaffold panel as a related or extended system rather than core dispersed-nanocomplex evidence. MDR, multidrug resistance; P-gp, P-glycoprotein; BCRP, breast cancer resistance protein.

A Tremella fuciformis polysaccharide-containing Pickering emulsion [97], for example, provides information on interfacial stabilization in a multicomponent system, whereas a chitosan nanoparticle/HA hydrogel [98] represents local-matrix delivery. In hydrogel- and scaffold-based formulations, the surrounding matrix may also contribute to local retention, cell adhesion, structural support, and tissue-regeneration cues. Because these effects arise from both the nanocomplex and the additional matrix components, the therapeutic outcome of the complete formulation cannot be assigned to the core polysaccharide–drug nanocomplex alone. To place these application areas and the related local-matrix systems in a common framework, the principal biomedical pathways are summarized schematically in Figure 3.

### 6.5. Manufacturing Challenges and Translational Considerations

Translation of polysaccharide–small-molecule supramolecular nanocomplexes depends not only on biological performance but also on whether formulation composition, processing conditions, and product quality can be reproduced during manufacture. For nanomedicines more broadly, poorly defined material attributes, batch variability, scale-up effects, and inadequate control of critical quality attributes remain important barriers to translation [67,99,100,101]. These problems are particularly relevant to polysaccharide-based systems because molecular-weight distribution, charge or substitution characteristics, impurity profile, and raw-material source may vary from batch to batch.

For solvent-exchange and microfluidic routes, phase ratio, supersaturation, mixing or flow conditions, concentration, temperature, and residence time can all influence particle formation. Solvent-removal conditions may further affect drug association and the free-drug fraction. During scale-up, these variables need to be linked to defined critical material attributes, critical process parameters, and critical quality attributes. Process equivalence can then be assessed using measurements such as particle size and PDI, drug content and free-drug fraction, and, where relevant, pH, osmolality, and residual solvent [67,99,100,101].

Sterilization and endotoxin control also need attention early in development, particularly for products intended for sterile administration. Autoclaving or irradiation may change polysaccharide structure or particle properties, whereas sterile filtration can lead to particle retention or adsorption. Aseptic manufacture may therefore be more suitable for sensitive formulations, provided that the relevant product quality attributes are maintained [102]. Endotoxin control is a separate issue from sterility. Raw polysaccharides, process water, and manufacturing equipment can all contribute to pyrogen contamination and require appropriate control [83,101].

Storage stability is best examined in the intended final dosage form because aggregation, drug leakage, degradation, freeze–thaw stress, and reorganization of weak interactions may change product performance over time. Lyophilization can improve long-term stability in some systems, but the choice of cryoprotectants or lyoprotectants and the drying cycle needs to be adapted to the specific formulation [103]. After reconstitution, redispersibility, particle size and PDI, drug content or free-drug fraction, potency, release behavior, residual moisture, and stability after defined storage provide useful measures of whether the original product properties have been recovered.

Container compatibility and route-specific quality requirements become increasingly important as a formulation approaches practical use. Storage and administration configurations may influence adsorption, particle shedding, extractables and leachables, colloidal properties, and container integrity [104]. Residual organic solvent also needs to be controlled when organic-solvent-based preparation methods are used. More broadly, reproducibility across polysaccharide lots depends on linking source, purification, molecular-weight distribution, composition, charge or substitution characteristics, and impurity profile to predefined nanocomplex quality attributes [67,99].

## 7. Conclusions

Bioactive polysaccharide–small-molecule drug supramolecular nanocomplexes combine reversible noncovalent assembly with measurable improvements in drug delivery, while the polysaccharide itself may also contribute biological effects in some systems. The current evidence is most developed for tumor-related applications, whereas studies in inflammatory diseases and wound healing are still less extensive. Molecular association, formulation performance, polysaccharide-related effects, receptor-mediated targeting, and pharmacological synergy represent different levels of evidence and should not be interpreted as equivalent. Related composites, hydrogels, and scaffolds can provide useful supporting information but fall outside the core nanocomplex category. Further development of these systems will require more quantitative evidence for carrier–drug interactions, better characterization and standardization of polysaccharides and their derivatives, clearer separation of carrier and drug contributions, longer-term safety assessment, and more reproducible manufacturing and quality control.

## Figures and Tables

**Table 1:** Representative interaction assignments, evidence strength, quantitative descriptors, and observed outcomes in bioactive polysaccharide–small-molecule drug supramolecular nanocomplexes.

Interaction	Representative System	Evidence Basis and Level	Quantitative Descriptors and Interpretation	Ref.
Hydrogen-bond-related association	Chitosan–prenylated flavonoid nanocomplexes (ICT, ICA, ICS).	^1^H NMR localized ICT 3-OH/7-OH involvement with chitosan amino groups; comparative flavonoid structures strengthen the assignment. FTIR and particle data are supportive/assembly-level evidence.	LC: ICT 16.29%, ICA 5.54%, ICS 6.58%. Supports structure-dependent hydrogen bonding together with electrostatic interactions. Acid/salt stability is an observed formulation outcome, not an effect attributable to hydrogen bonding alone.	[20]
	Curdlan complexes with curcumin, quercetin, or chlorogenic acid.	FTIR, XRD, DSC and pH-driven assembly are consistent with combined hydrogen bonding and hydrophobic association; no single-force thermodynamic isolation was reported.	Improved physicochemical/GI stability, antioxidant activity and prebiotic-related outcomes were observed. These endpoints are reported as whole-formulation effects; direct causality from an individual interaction was not established.	[21]
Hydrophobic association	Stearic-acid-modified Bletilla striata polysaccharide micelles loaded with docetaxel.	Chemical amphiphilization and self-assembly are supported by structural characterization; DLS/TEM, loading and release are assembly/outcome evidence rather than direct proof of one force.	DS 12.94%; DTX/carrier 1:6 (*w*/*w*); size 125.30 ± 1.89 nm; EE 86.6 ± 0.17%; LC 14.8 ± 0.13%; 48-h release 66.93 ± 1.79% versus 97.06 ± 1.56% for free-drug formulation.	[22]
	Octadecanoate oat β-glucan micelles loaded with myricetin.	Pyrene fluorescence gives a quantitative self-association threshold; FTIR/XRD and DLS/TEM provide complementary incorporation and assembly evidence.	DS 0.057; CMC 59.4 μg/mL. CMC supports hydrophobic self-association, whereas improved solubility/retention and antioxidant activity are downstream formulation outcomes.	[23]
Electrostatic organization	Related multicomponent fucoidan/PEI nanoparticles bearing doxorubicin.	Charge complementarity and zeta-potential/particle changes support electrostatic organization between fucoidan and PEI. DLS and zeta potential do not constitute molecular proof of polysaccharide–drug binding.	Used only as a multicomponent electrostatic-assembly example; antitumor outcome cannot be assigned specifically to a fucoidan–DOX molecular interaction.	[24]
	One-pot hyaluronate/doxorubicin nanoaggregates.	Known opposite charges, component-ratio dependence and composition analysis support electrostatic involvement. Cation–π association was proposed by the authors; DLS/turbidity describe assembly rather than interaction strength.	[DOX]/[HA] = 1: negligible aggregation; ratio 2: 7825 ± 1060 nm, PDI 2.913; optimized ratio 5: ~250 nm. Drug content ~0.24 mg DOX/mg aggregate, corresponding to ~3.5 DOX per HA chain.	[25]
π–π-related aromatic association	Related CS-g-PZLL micelles co-delivering doxorubicin and p53; PZLL contains aromatic carbobenzyloxy groups.	Fluorescence spectroscopy supported DOX–micelle aromatic association; ^1^H NMR/FTIR characterized the carrier. Because the system is multicomponent, the evidence is not treated as core binary proof.	DOX loading amount 12.8%. Direct π–π interaction is plausible because the modified carrier contains aromatic groups, but fluorescence alone cannot fully separate π–π stacking from hydrophobic partitioning.	[26]
	Chitosan/doxorubicin encapsulation and release model.	Molecular-dynamics simulation distinguished DOX–DOX aromatic stacking from chitosan–DOX interactions and showed dependence on chitosan protonation state.	No single transferable binding value is used because interaction energies depend on protonation state and configuration. The model supports drug–drug π–π stacking rather than direct native-chitosan–DOX π–π binding.	[27]

**Table 2:** Comparison of preparation methods for polysaccharide–small-molecule supramolecular nanocomplexes.

Preparation Method	Polysaccharide Type	Interaction Type	Advantages	Disadvantages
Nanoprecipitation/solvent exchange Solvent/CPPs: water-miscible organic phase + aqueous antisolvent; phase ratio, concentration, addition/flow, mixing, solvent removal.	Amphiphilic or hydrophobically modified polysaccharides; suitable for poorly water-soluble drugs. HA-PCL/DOX: 146.2 ± 9.6 nm; encapsulation 54.9 ± 4.0%; loading 3.6 ± 0.4% [35].	Solvent-induced desolvation and nucleation, usually coupled with hydrophobic association.	Simple and rapid; useful for hydrophobic payloads; particle formation can be tuned by mixing and solvent exchange; continuous/controlled mixing can improve reproducibility.	Organic-solvent residue and local supersaturation; sensitive to mixing history; scale-up must preserve mass-transfer conditions; filtration/aseptic or terminal sterilization requires formulation-specific validation.
Polyelectrolyte complexation Solvent/CPPs: mainly aqueous; pH, ionic strength, charge ratio, concentration, order of addition, mixing.	Ionizable polysaccharides (e.g., chitosan, alginate, sulfate/carboxyl-containing polysaccharides) and oppositely charged partners. Chitosan-alginate/DOX: ~100 nm; encapsulation ~95% [36].	Electrostatic complexation with possible hydrogen-bond and hydrophobic contributions.	Mild aqueous processing; low organic-solvent burden; useful for charge-driven assembly and lab-scale formulation screening.	Highly sensitive to pH, salt and stoichiometry; charge neutralization may cause aggregation/precipitation; batch reproducibility depends on addition order and mixing; sterilization may alter charge balance or polysaccharide integrity.
Direct aqueous self-assembly Solvent/CPPs: water-dominant; carrier amphiphilicity, concentration, substitution, drug/carrier ratio, pH, temperature, mixing/energy input.	Native amphiphilic or amphiphilically modified polysaccharides. Turmeric-pomace polysaccharide/curcumin: 174.93 ± 3.45 nm; PDI 0.31; encapsulation 40.03 ± 0.54%; loading 15.39 ± 0.21 μg/mg [37].	Hydrophobic association, hydrogen bonding and other weak forces depending on the carrier/drug pair.	Simple carrier assembly; potentially low-solvent processing; avoids rapid antisolvent precipitation; useful when the polysaccharide already provides suitable amphiphilicity.	Not applicable to every carrier/drug pair; poorly soluble drugs may still require limited co-solvent; insufficient amphiphilicity lowers loading, whereas excessive modification/concentration promotes aggregation; reproducibility depends on substitution pattern and molecular heterogeneity.
Microfluidic-assisted assembly Solvent/CPPs: aqueous or solvent-exchange streams; flow-rate ratio, total flow, channel geometry, residence time, concentration and solvent composition.	Polysaccharides compatible with controlled solvent exchange or ionic complexation. Chitosan/TPP-curcumin: 157 ± 41 nm; PDI 0.31 ± 0.02; encapsulation 74.81 ± 2.78% [38].	Parent assembly mechanism (electrostatic or hydrophobic), with microscale mixing controlling nucleation and particle growth.	Precise and tunable mixing; lower batch-to-batch variability; reproducible microscale operation; suitable for high-throughput formulation screening and scale-out strategies.	Device sterilization, sterile connections and cleaning validation remain necessary; viscosity, clogging, pressure drop and material/solvent compatibility limit throughput; scale-out is usually more realistic than simple geometric scale-up.

**Table 3:** Comparison of characterization methods and evidence levels for polysaccharide–small-molecule supramolecular nanocomplexes.

Characterization Method	Evidence Type	Information Obtained	Advantages	Disadvantages
FTIR/conventional NMR/UV–vis/fluorescence [49]	Molecular/spectroscopic	Functional-group changes, chemical microenvironment and possible carrier–drug association; NMR chemical shifts/relaxation and optical shifts or quenching provide complementary molecular-level evidence.	Widely accessible; low sample demand; useful for comparing free components, physical mixtures and assembled systems.	Mostly indirect for interaction assignment; peak overlap, water bands, inner-filter effects and scattering are important for heterogeneous polysaccharides; should not be used alone to prove a dominant interaction.
DOSY-NMR [50]	Molecular mobility/association	Diffusion coefficients and co-diffusion behavior; can help distinguish free and associated species and examine molecular localization/exchange in solution.	Solution-state information without drying; adds a mobility dimension to conventional NMR.	Broad/overlapping polysaccharide signals and multiple size populations complicate fitting; requires adequate solubility and spectral resolution.
ITC/SPR [51,52]	Quantitative affinity/thermodynamics	ITC: binding constant, stoichiometry, ΔH and derived ΔG/ΔS. SPR: real-time association/dissociation and affinity when immobilization is feasible.	Provides quantitative interaction parameters that conventional spectra cannot supply; useful for testing mechanistic hypotheses.	ITC needs sufficient heat signal and a tractable binding model; SPR requires immobilization and may suffer from mass-transfer/surface artifacts. Heterogeneous polysaccharides can yield non-single-site behavior.
DLS/ζ-potential/NTA [53,54]	Colloidal/assembly	Hydrodynamic size (z-average), PDI and electrokinetic behavior; NTA provides number-based size distribution and particle concentration.	Rapid solution-state assessment of particle formation, aggregation and batch consistency; NTA provides a useful orthogonal single-particle measurement.	DLS is intensity-weighted and highly sensitive to large particles; it does not measure molecular interactions. Report temperature, dispersant/pH/ionic strength/viscosity, concentration/dilution, instrument configuration, replicates, z-average/PDI and distribution basis. ζ-potential depends on medium and conversion model; no fixed |ζ| threshold is universally valid.
TEM/cryo-TEM [55]	Morphology/nanostructure	Particle morphology, approximate size and internal/core–shell features; cryo-TEM better preserves hydrated soft structures.	Direct visualization; cryogenic preparation reduces drying/staining artifacts compared with conventional TEM.	Small field of view and possible sampling bias; conventional TEM may distort soft polysaccharide assemblies, while cryo-TEM is technically demanding and resource intensive.
XRD/DSC/TGA [49]	Solid-state/thermal	Crystallinity, thermal transitions and degradation/water/residual-solvent behavior; useful for assessing changes in detectable drug physical state.	Complements colloidal data and helps distinguish crystalline-drug retention from reduced crystallinity or amorphous dispersion.	Disappearance of drug peaks/endotherms does not by itself prove complete amorphization or molecular dispersion; low drug loading, matrix dilution and peak overlap can mask signals.
SAXS/SANS [56]	Solution/internal structure	Quantitative size, shape and internal organization over nanometer-length scales; model-derived structural parameters.	Ensemble solution-state structural information; useful for soft particles that are sensitive to drying.	Interpretation is model-dependent; polydispersity and compositional heterogeneity can produce non-unique fits; SANS access is limited.
SEC-MALS/analytical ultracentrifugation [57]	Polysaccharide heterogeneity/hydrodynamics	Molecular-weight distribution, molar mass, size/conformation and sedimentation behavior; helps characterize the carrier before linking structure to assembly.	Particularly useful for defining heterogeneous natural polysaccharides and batch differences rather than treating the carrier as a single molecular species.	Requires appropriate separation/solvent conditions and concentration control; aggregation, non-ideal interactions and broad distributions complicate analysis.
Molecular docking/MD [58]	Computational/mechanistic	Candidate binding sites/orientations, hydrogen-bond persistence, interaction energies, RMSD/RMSF and dynamic conformational stability.	Connects experimental observations with atomistic hypotheses and allows controlled comparison of interaction scenarios.	Strongly dependent on carbohydrate model, force field, ionization, substitution pattern and simulation time; simplified models cannot fully reproduce natural-polysaccharide heterogeneity.

**Table 4:** Quantitative comparison of representative biomedical applications of core bioactive polysaccharide–small-molecule drug supramolecular nanocomplexes.

System	Preparation	Size/PDI/ζ	Loading/Release	Model/Route	Quantitative Outcome	Limitation
[Tumor] Stearic-acid-grafted BSP glucomannan (DS 12.94%; CAC 3.09 μg/mL); docetaxel; 2% mannitol [86].	Micellar self-assembly; ethanol-assisted loading, solvent evaporation and lyophilization.	97.01 ± 3.17 nm; PDI NR; ζ = −19.56 ± 0.22 mV.	EE 81.11 ± 0.18%; DL 9.13 ± 0.17%. Release: dialysis, PBS pH 7.4 + 0.2% Tween 80, 37 °C; 66.93 ± 1.79% at 48 h vs. ~100% from injection.	HeLa/HepG2; Wistar rats; i.v. 20 mg/kg.	AUC0–∞ 65.39 ± 5.21 vs. 47.73 ± 0.49 μg·h/mL; absolute bioavailability 1.39-fold higher.	Chemically modified BSP; in vivo evidence mainly pharmacokinetic rather than tumor-bearing efficacy.
[Tumor] BSP–vitamin E succinate amphiphile (CMC 48.3 μg/mL); andrographolide; vitamin E succinate modifier [87].	Dialysis; drug introduced from ethanol into aqueous BSP-VES micelles.	84.68 ± 2.08 nm; PDI 0.241 ± 0.006; ζ = −12.49 ± 2.28 mV.	EE 91.15 ± 0.05%; DL 6.84 ± 0.01%. Release: 3.5-kDa dialysis, PBS pH 7.4, 37 °C, 100 rpm; 47.6% at 24 h; free drug almost fully released within 8 h.	CT26/NCM460; subcutaneous and orthotopic CT26(-Luc) BALB/c mice; i.v. 10 mg/kg for imaging.	CT26 IC50 11.69 vs. 31.16 μg/mL for free drug; 24-h tumor accumulation 55.37% ID/g (subcutaneous) and 17.31% ID/g (orthotopic).	VES-modified BSP; receptor-specific blocking and long-term efficacy/safety evidence remain limited.
[Tumor] HA–deoxycholic-acid derivative (HD9; 9.4 DOCA groups/HA molecule); doxorubicin; triethylamine used during loading [88].	Dialysis followed by lyophilization and sonication.	212 ± 23.1 nm; PDI 0.300; ζ NR (HD9, 5 wt% drug feed).	Loading efficiency 99.33 ± 10.91%; DL 4.97 ± 0.57%. Release: dialysis in PBS pH 7.2, 37 °C, 100 rpm; initial burst followed by slower release.	HeLa cells; no animal administration.	Higher DOCA substitution reduced particle size and increased loading efficiency; HD9 at 5 wt% DOX feed gave ~99.3% loading efficiency.	In vitro only; particle attributes varied with substitution degree and receptor-mediated targeting was not established.
[Tumor] Native levan from Erwinia herbicola (~1 MDa); paclitaxel; no additional carrier modifier [89].	One-pot nanoprecipitation driven by levan–paclitaxel association.	Size, PDI and ζ NR in the accessible primary-study summary.	EE 98.7%; DL NR. Release: physiological buffer containing surfactant; sustained release reported with long-term colloidal stability.	SCC7 cells and SCC7 tumor-bearing mice; systemic administration.	Tumor accumulation 3.7-fold higher than free dye, with improved PTX antitumor activity.	Several formulation/release parameters were incompletely reported; targeting evidence remains model-specific.
[Inflammation] Dextran sulfate (36–50 kDa) bearing aminoethyl 5β-cholanic acid; methotrexate [90].	Self-assembly with methotrexate loading by dialysis.	220 nm; PDI and ζ NR.	Loading efficiency 73.0%; DL NR. Detailed release conditions NR in the accessible primary-study summary.	Activated RAW264.7 macrophages; collagen-induced arthritis mice; systemic/tail-vein administration.	Inflamed-joint accumulation ~12-fold higher than in wild-type mice; efficacy exceeded free MTX.	Several formulation/release metrics were not reported; evidence is confined to the CIA model.
[Wound] Oligochitosan (MW 2.7 kDa; 80% deacetylation); curcumin; no additional carrier component [91].	Drug–polyelectrolyte complexation; pH, salt, CUR concentration and charge ratio optimized.	~140 nm; PDI NR at optimum; ζ ~+20 mV.	CUR payload ~78% (*w*/*w*); utilization ~93%; production yield ~42%. No conventional membrane-release test; supersaturation in PBS at 37 °C and stability up to 72 h were evaluated.	HaCaT cells; full-thickness dorsal wounds in male mice; 200 μL topical administration.	At 0.05 mg/mL CUR, residual wound area was 8 ± 1% (day 7) and 2 ± 0.6% (day 9) vs. 15 ± 1% and 8 ± 1% for native CUR.	Topical wound model only; conventional release kinetics and long-term safety were not evaluated.

## Data Availability

The data and literature resources used in this review are available from the published references listed in this article.

## Abbreviations

The following abbreviations are used in this manuscript:

APS	Astragalus polysaccharide
ASGPR	asialoglycoprotein receptor
AUC	area under the curve
AZM	azithromycin
BCP	biphasic calcium phosphate
BSP	Bletilla striata polysaccharide
CAC	critical aggregation concentration
Cal	calcitriol
CD44	cluster of differentiation 44
CMC	critical micelle concentration
Cmax	maximum plasma concentration
CRediT	Contributor Roles Taxonomy
CS	chitosan
Cu	copper
Cur	curcumin
DLS	dynamic light scattering
DOX	doxorubicin
DSC	differential scanning calorimetry
ECM	extracellular matrix
FTIR	Fourier transform infrared spectroscopy
GLUT	glucose transporter
HA	hyaluronic acid
MD	molecular dynamics
MDR	multidrug resistance
MMP	matrix metalloproteinase
MMPs	matrix metalloproteinases
NDDSs	nanodrug delivery systems
NMR	nuclear magnetic resonance
NPs	nanoparticles
OA	osteoarthritis
PDI	polydispersity index
PEI	polyethyleneimine
PTX	paclitaxel
QbD	Quality by Design
RMSD	root-mean-square deviation
RMSF	root-mean-square fluctuation
ROS	reactive oxygen species
TCMPs	traditional Chinese medicine polysaccharides
TEM	transmission electron microscopy
TG-DSC	thermogravimetry–differential scanning calorimetry
TGA	thermogravimetric analysis
Tmax	time to maximum plasma concentration
UV–vis	ultraviolet–visible
XRD	X-ray diffraction
cryo-TEM	cryogenic transmission electron microscopy
DOSY	diffusion-ordered spectroscopy
ITC	isothermal titration calorimetry
NTA	nanoparticle tracking analysis
SANS	small-angle neutron scattering
SAXS	small-angle X-ray scattering
SEC-MALS	size-exclusion chromatography coupled with multi-angle light scattering
SPR	surface plasmon resonance

## References

[1] Liu Y., Xu J., Guo Y. (2025). Natural polysaccharide-based nano-drug delivery systems: Innovative strategies and research advances in cancer therapy—A review. Int. J. Biol. Macromol..

[2] Han X., Gong C., Yang Q., Zheng K., Wang Z., Zhang W. (2024). Biomimetic Nano-Drug Delivery System: An Emerging Platform for Promoting Tumor Treatment. Int. J. Nanomed..

[3] Hu Q., Lu Y., Luo Y. (2021). Recent advances in dextran-based drug delivery systems: From fabrication strategies to applications. Carbohydr. Polym..

[4] Plucinski A., Lyu Z., Schmidt B. (2021). Polysaccharide nanoparticles: From fabrication to applications. J. Mater. Chem. B.

[5] Gong H., Li W., Sun J., Jia L., Guan Q., Guo Y., Wang Y. (2022). A review on plant polysaccharide based on drug delivery system for construction and application, with emphasis on traditional Chinese medicine polysaccharide. Int. J. Biol. Macromol..

[6] Maity P., Sen I.K., Chakraborty I., Mondal S., Bar H., Bhanja S.K., Mandal S., Maity G.N. (2021). Biologically active polysaccharide from edible mushrooms: A review. Int. J. Biol. Macromol..

[7] Yu Y., Shen M., Song Q., Xie J. (2018). Biological activities and pharmaceutical applications of polysaccharide from natural resources: A review. Carbohydr. Polym..

[8] Ma Y., Morozova S.M., Kumacheva E. (2024). From Nature-Sourced Polysaccharide Particles to Advanced Functional Materials. Adv. Mater..

[9] Li M., Zhao Y., Zhang W., Zhang S., Zhang S. (2021). Multiple-therapy strategies via polysaccharides-based nano-systems in fighting cancer. Carbohydr. Polym..

[10] Wu Z., Li H., Zhao X., Ye F., Zhao G. (2022). Hydrophobically modified polysaccharides and their self-assembled systems: A review on structures and food applications. Carbohydr. Polym..

[11] Geng W.C., Jiang Z.T., Chen S.L., Guo D.S. (2024). Supramolecular interaction in the action of drug delivery systems. Chem. Sci..

[12] Li T., Wang Q., Rui C., Ren L., Dai M., Bi Y., Yang Y. (2025). Targeted isolation and AI-based analysis of edible fungal polysaccharides: Emphasizing tumor immunological mechanisms and future prospects as mycomedicines. Int. J. Biol. Macromol..

[13] Wang B., Wang X., Xiong Z., Lu G., Ma W., Lv Q., Wang L., Jia X., Feng L. (2022). A review on the applications of Traditional Chinese medicine polysaccharides in drug delivery systems. Chin. Med..

[14] Kong F., Chen T., Li X., Jia Y. (2021). The Current Application and Future Prospects of Astragalus Polysaccharide Combined With Cancer Immunotherapy: A Review. Front. Pharmacol..

[15] Wang J., Wu X., Chen J., Gao T., Zhang Y., Yu N. (2024). Traditional Chinese medicine polysaccharide in nano-drug delivery systems: Current progress and future perspectives. Biomed. Pharmacother..

[16] Pepe G., Calce E., Verdoliva V., Saviano M., Maglione V., Di Pardo A., De Luca S. (2020). Curcumin-Loaded Nanoparticles Based on Amphiphilic Hyaluronan-Conjugate Explored as Targeting Delivery System for Neurodegenerative Disorders. Int. J. Mol. Sci..

[17] Zeng Y., Xiang Y., Sheng R., Tomás H., Rodrigues J., Gu Z., Zhang H., Gong Q., Luo K. (2021). Polysaccharide-based nanomedicines for cancer immunotherapy: A review. Bioact. Mater..

[18] Zhang Y., Zhang Y., Ding R., Zhang K., Guo H., Lin Y. (2024). Self-Assembled Nanocarrier Delivery Systems for Bioactive Compounds. Small.

[19] Zhao W., Huang W., Wu X., Li X., Gao W., Guo L. (2026). Natural small molecule-polysaccharide nanoparticles: Preparation, characteristics-efficacy modulation, and drug delivery applications. Carbohydr. Polym..

[20] Wang J., Chen J., Jiang Y., Yang B., Wen L. (2025). Effect of phenolic hydrogen on the formation of chitosan -prenylated flavonoids nanocomplexes. Food Hydrocoll..

[21] Li H., Xu S., Xie Y., Zhang Q., Ding S., Wang R., Fu F., Zhan X. (2024). Curdlan-polyphenol complexes prepared by pH-driven effectively enhanced their physicochemical stability, antioxidant and prebiotic activities. Int. J. Biol. Macromol..

[22] Guan Q., Sun D., Zhang G., Sun C., Wang M., Ji D., Yang W. (2016). Docetaxel-Loaded Self-Assembly Stearic Acid-Modified Bletilla striata Polysaccharide Micelles and Their Anticancer Effect: Preparation, Characterization, Cellular Uptake and In Vitro Evaluation. Molecules.

[23] Yang W., Guo L., Li F., Liu X., Nie S., Xie M., Huang D. (2019). Hydrophobically Modified Glucan as an Amphiphilic Carbohydrate Polymer for Micellar Delivery of Myricetin. Molecules.

[24] Pawar V.K., Singh Y., Sharma K., Shrivastav A., Sharma A., Singh A., Meher J.G., Singh P., Raval K., Kumar A. (2019). Improved chemotherapy against breast cancer through immunotherapeutic activity of fucoidan decorated electrostatically assembled nanoparticles bearing doxorubicin. Int. J. Biol. Macromol..

[25] Lim Y.G., Park H.G., Park K. (2025). Facile One-Pot Preparation of Self-Assembled Hyaluronate/Doxorubicin Nanoaggregates for Cancer Therapy. Biomimetics.

[26] Wang G.-H., Yang H.-K., Zhao Y., Zhang D.-W., Zhang L.-M., Lin J.-T. (2015). Codelivery of doxorubicin and p53 by biodegradable micellar carriers based on chitosan derivatives. RSC Adv..

[27] Li J., Ying S., Ren H., Dai J., Zhang L., Liang L., Wang Q., Shen Q., Shen J.W. (2020). Molecular dynamics study on the encapsulation and release of anti-cancer drug doxorubicin by chitosan. Int. J. Pharm..

[28] Fan Y., Liu Y., Wu Y., Dai F., Yuan M., Wang F., Bai Y., Deng H. (2021). Natural polysaccharides based self-assembled nanoparticles for biomedical applications—A review. Int. J. Biol. Macromol..

[29] Yang X., Shi X., D’Arcy R., Tirelli N., Zhai G. (2018). Amphiphilic polysaccharides as building blocks for self-assembled nanosystems: Molecular design and application in cancer and inflammatory diseases. J. Control. Release.

[30] Dubashynskaya N.V., Gasilova E.R., Skorik Y.A. (2023). Nano-Sized Fucoidan Interpolyelectrolyte Complexes: Recent Advances in Design and Prospects for Biomedical Applications. Int. J. Mol. Sci..

[31] Quinones J.P., Jokinen J., Keinänen S., Covas C.P., Brüggemann O., Ossipov D. (2018). Self-assembled hyaluronic acid-testosterone nanocarriers for delivery of anticancer drugs. Eur. Polym. J..

[32] Abbasi Y.F., Bera H., Cun D., Yang M. (2023). Recent advances in pH/enzyme-responsive polysaccharide-small-molecule drug conjugates as nanotherapeutics. Carbohydr. Polym..

[33] Garcia-Jimenez A., Roman-Guerrero A., Perez-Alonso C., Fouconnier B. (2022). Liquid-liquid and liquid-solid separation in self-assembled chitosan-alginate and chitosan-pectin complexes. Int. J. Biol. Macromol..

[34] Jiang Y., Yan C., Li M., Chen S., Chen Z., Yang L., Luo K. (2024). Delivery of natural products via polysaccharide-based nanocarriers for cancer therapy: A review on recent advances and future challenges. Int. J. Biol. Macromol..

[35] Shahriari M., Taghdisi S.M., Abnous K., Ramezani M., Alibolandi M. (2019). Synthesis of hyaluronic acid-based polymersomes for doxorubicin delivery to metastatic breast cancer. Int. J. Pharm..

[36] Katuwavila N.P., Perera A.D.L.C., Samarakoon S.R., Soysa P., Karunaratne V., Amaratunga G.A.J., Karunaratne D.N. (2016). Chitosan-Alginate Nanoparticle System Efficiently Delivers Doxorubicin to MCF-7 Cells. J. Nanomater..

[37] Yin B., Li L., Wang P., Zhu W., Bu Y., Li L., Zhang Z., Zheng Y., Zheng Z. (2025). Structural Characterization and Functional Properties of Turmeric Pomace Polysaccharides-Curcumin Nanoparticles. Food Chem. Int..

[38] Chiesa E., Greco A., Riva F., Tosca E.M., Dorati R., Pisani S., Modena T., Conti B., Genta I. (2019). Staggered Herringbone Microfluid Device for the Manufacturing of Chitosan/TPP Nanoparticles: Systematic Optimization and Preliminary Biological Evaluation. Int. J. Mol. Sci..

[39] Kuddushi M., Kanike C., Xu B.B., Zhang X. (2025). Recent advances in nanoprecipitation: From mechanistic insights to applications in nanomaterial synthesis. Soft Matter.

[40] Luque-Alcaraz A.G., Lizardi-Mendoza J., Goycoolea F.M., Higuera-Ciapara I., Argüelles-Monal W. (2016). Preparation of chitosan nanoparticles by nanoprecipitation and their ability as a drug nanocarrier. RSC Adv..

[41] Hamman J.H. (2010). Chitosan based polyelectrolyte complexes as potential carrier materials in drug delivery systems. Mar. Drugs.

[42] Luo Y., Wang Q. (2014). Recent development of chitosan-based polyelectrolyte complexes with natural polysaccharides for drug delivery. Int. J. Biol. Macromol..

[43] Potaś J., Szymańska E., Winnicka K. (2020). Challenges in developing of chitosan–Based polyelectrolyte complexes as a platform for mucosal and skin drug delivery. Eur. Polym. J..

[44] Hassani L.N., Hendra F., Bouchemal K. (2012). Auto-associative amphiphilic polysaccharides as drug delivery systems. Drug Discov. Today.

[45] Zhu J., Guo X., Guo T., Yang Y., Cui X., Pan J., Qu Y., Wang C. (2018). Novel pH-responsive and self-assembled nanoparticles based on Bletilla striata polysaccharide: Preparation and characterization. RSC Adv..

[46] Chiesa E., Dorati R., Pisani S., Conti B., Bergamini G., Modena T., Genta I. (2018). The Microfluidic Technique and the Manufacturing of Polysaccharide Nanoparticles. Pharmaceutics.

[47] Siavashy S., Soltani M., Ghorbani-Bidkorbeh F., Fallah N., Farnam G., Mortazavi S.A., Shirazi F.H., Tehrani M.H.H., Hamedi M.H. (2021). Microfluidic platform for synthesis and optimization of chitosan-coated magnetic nanoparticles in cisplatin delivery. Carbohydr. Polym..

[48] Tomeh M.A., Zhao X. (2020). Recent Advances in Microfluidics for the Preparation of Drug and Gene Delivery Systems. Mol. Pharm..

[49] Mourdikoudis S., Pallares R.M., Thanh N.T.K. (2018). Characterization techniques for nanoparticles: Comparison and complementarity upon studying nanoparticle properties. Nanoscale.

[50] Schlattmann D., Weber B., Wyszynski L., Schönhoff M., Haas H. (2024). Molecular localization and exchange kinetics in pharmaceutical liposome and mRNA lipoplex nanoparticle products determined by small angle X-ray scattering and pulsed field gradient NMR diffusion measurements. Eur. J. Pharm. Biopharm..

[51] Cavalcanti I.D.L., Xavier Junior F.H., Magalhães N.S.S., Nogueira M.C.B.L. (2023). Isothermal titration calorimetry (ITC) as a promising tool in pharmaceutical nanotechnology. Int. J. Pharm..

[52] Chain C.Y., Daza Millone M.A., Cisneros J.S., Ramirez E.A., Vela M.E. (2021). Surface Plasmon Resonance as a Characterization Tool for Lipid Nanoparticles Used in Drug Delivery. Front. Chem..

[53] Bhattacharjee S. (2016). DLS and zeta potential–What they are and what they are not?. J. Control. Release.

[54] Krueger A.B., Carnell P., Carpenter J.F. (2016). Characterization of Factors Affecting Nanoparticle Tracking Analysis Results with Synthetic and Protein Nanoparticles. J. Pharm. Sci..

[55] Kuntsche J., Horst J.C., Bunjes H. (2011). Cryogenic transmission electron microscopy (cryo-TEM) for studying the morphology of colloidal drug delivery systems. Int. J. Pharm..

[56] Thakral S., Kim K. (2021). Small-angle scattering for characterization of pharmaceutical materials. TrAC Trends Anal. Chem..

[57] Morris G.A., Adams G.G., Harding S.E. (2014). On hydrodynamic methods for the analysis of the sizes and shapes of polysaccharides in dilute solution: A short review. Food Hydrocoll..

[58] Xu X., Liu A., Liu S., Ma Y., Zhang X., Zhang M., Zhao J., Sun S., Sun X. (2023). Application of molecular dynamics simulation in self-assembled cancer nanomedicine. Biomater. Res..

[59] Nan Z., Chen L., Li G., Li H., Li Y., Ma J., Ding J., Yang J. (2025). A method for the quantitative analysis of Lycium barbarum polysaccharides (LBPs) using Fourier-transform infrared spectroscopy (FTIR): From theoretical computation to experimental application. Spectrochim. Acta A Mol. Biomol. Spectrosc..

[60] Yao H.-Y.-Y., Wang J.-Q., Yin J.-Y., Nie S.-P., Xie M.-Y. (2021). A review of NMR analysis in polysaccharide structure and conformation: Progress, challenge and perspective. Food Res. Int..

[61] Zhang C., Jin Z., Zeng B., Wang W., Palui G., Mattoussi H. (2020). Characterizing the Brownian Diffusion of Nanocolloids and Molecular Solutions: Diffusion-Ordered NMR Spectroscopy vs Dynamic Light Scattering. J. Phys. Chem. B.

[62] Archer W.R., Schulz M.D. (2020). Isothermal titration calorimetry: Practical approaches and current applications in soft matter. Soft Matter.

[63] Zhang J., Liu B., Chen H., Zhang L., Jiang X. (2024). Application and Method of Surface Plasmon Resonance Technology in the Preparation and Characterization of Biomedical Nanoparticle Materials. Int. J. Nanomed..

[64] Rizvi A., Mulvey J.T., Carpenter B.P., Talosig R., Patterson J.P. (2021). A Close Look at Molecular Self-Assembly with the Transmission Electron Microscope. Chem. Rev..

[65] Carvalho P.M., Felício M.R., Santos N.C., Gonçalves S., Domingues M.M. (2018). Application of Light Scattering Techniques to Nanoparticle Characterization and Development. Front. Chem..

[66] Gericke M., Schulze P., Heinze T. (2020). Nanoparticles Based on Hydrophobic Polysaccharide Derivatives-Formation Principles, Characterization Techniques, and Biomedical Applications. Macromol. Biosci..

[67] Đorđević S., Gonzalez M.M., Conejos-Sánchez I., Carreira B., Pozzi S., Acúrcio R.C., Satchi-Fainaro R., Florindo H.F., Vicent M.J. (2022). Current hurdles to the translation of nanomedicines from bench to the clinic. Drug Deliv. Transl. Res..

[68] Matson J.B., Steele A.Q., Mase J.D., Schulz M.D. (2024). Polymer characterization by size-exclusion chromatography with multi-angle light scattering (SEC-MALS): A tutorial review. Polym. Chem..

[69] Khezri A., Karimi A., Yazdian F., Jokar M., Mofradnia S.R., Rashedi H., Tavakoli Z. (2018). Molecular dynamic of curcumin/chitosan interaction using a computational molecular approach: Emphasis on biofilm reduction. Int. J. Biol. Macromol..

[70] Fadda E., Woods R.J. (2010). Molecular simulations of carbohydrates and protein-carbohydrate interactions: Motivation, issues and prospects. Drug Discov. Today.

[71] Du B., Nie S., Peng F., Yang Y., Xu B. (2022). A narrative review on conformational structure characterization of natural polysaccharides. Food Front..

[72] Baltz N., Valentin L., Scherließ R. (2026). How to determine entrapment efficiency in lipid and polymeric nanoparticles: A methodological guide. OpenNano.

[73] Liu M., Zhang G., Zhou K., Wen J., Zheng F., Sun L., Ren X. (2023). Structural characterization, antioxidant activity, and the effects of Codonopsis pilosula polysaccharides on the solubility and stability of flavonoids. J. Pharm. Biomed. Anal..

[74] Liu F., Sun L., You G., Liu H., Ren X., Wang M. (2020). Effects of Astragalus polysaccharide on the solubility and stability of 15 flavonoids. Int. J. Biol. Macromol..

[75] Nguyen M.H., Yu H., Kiew T.Y., Hadinoto K. (2015). Cost-effective alternative to nano-encapsulation: Amorphous curcumin-chitosan nanoparticle complex exhibiting high payload and supersaturation generation. Eur. J. Pharm. Biopharm..

[76] Wong J.J.L., Yu H., Hadinoto K. (2018). Examining practical feasibility of amorphous curcumin-chitosan nanoparticle complex as solubility enhancement strategy of curcumin: Scaled-up production, dry powder transformation, and long-term physical stability. Colloids Surf. A Physicochem. Eng. Asp..

[77] Barclay T.G., Day C.M., Petrovsky N., Garg S. (2019). Review of polysaccharide particle-based functional drug delivery. Carbohydr. Polym..

[78] Qiu A., Wang Y., Zhang G., Wang H. (2022). Natural Polysaccharide-Based Nanodrug Delivery Systems for Treatment of Diabetes. Polymers.

[79] Hsu C.Y., Allela O.Q.B., Hussein A.M., Mustafa M.A., Kaur M., Alaraj M., Al-Hussainy A.F., Radi U.K., Ubaid M., Idan A.H. (2024). Recent advances in polysaccharide-based drug delivery systems for cancer therapy: A comprehensive review. Artif. Cells Nanomed. Biotechnol..

[80] Yu H., Nguyen M.H., Hadinoto K. (2018). Effects of chitosan molecular weight on the physical and dissolution characteristics of amorphous curcumin-chitosan nanoparticle complex. Drug Dev. Ind. Pharm..

[81] Modi S., Anderson B.D. (2013). Determination of drug release kinetics from nanoparticles: Overcoming pitfalls of the dynamic dialysis method. Mol. Pharm..

[82] Il’ina A.V., Varlamov V.P. (2016). Determination of residual protein and endotoxins in chitosan (review). Appl. Biochem. Microbiol..

[83] Li Y., Fujita M., Boraschi D. (2017). Endotoxin Contamination in Nanomaterials Leads to the Misinterpretation of Immunosafety Results. Front. Immunol..

[84] Hannon G., Lysaght J., Liptrott N.J., Prina-Mello A. (2019). Immunotoxicity Considerations for Next Generation Cancer Nanomedicines. Adv. Sci..

[85] Vlot A.H.C., Aniceto N., Menden M.P., Ulrich-Merzenich G., Bender A. (2019). Applying synergy metrics to combination screening data: Agreements, disagreements and pitfalls. Drug Discov. Today.

[86] Guan Q., Zhang G., Sun D., Wang Y., Liu K., Wang M., Sun C., Zhang Z., Li B., Lv J. (2017). In vitro and in vivo evaluation of docetaxel-loaded stearic acid-modified Bletilla striata polysaccharide copolymer micelles. PLoS ONE.

[87] Yue Z., Zhu Y., Chen T., Feng T., Zhou Y., Zhang J., Zhang N., Yang J., Luo G., Wang Z. (2024). Bletilla striata polysaccharide-coated andrographolide nanomicelles for targeted drug delivery to enhance anti-colon cancer efficacy. Front. Immunol..

[88] Wei W.H., Dong X.M., Liu C.G. (2015). In Vitro Investigation of Self-Assembled Nanoparticles Based on Hyaluronic Acid-Deoxycholic Acid Conjugates for Controlled Release Doxorubicin: Effect of Degree of Substitution of Deoxycholic Acid. Int. J. Mol. Sci..

[89] Lee J.S., Park E., Oh H., Choi W.I., Koo H. (2023). Levan nanoparticles with intrinsic CD44-targeting ability for tumor-targeted drug delivery. Int. J. Biol. Macromol..

[90] Heo R., You D.G., Um W., Choi K.Y., Jeon S., Park J.S., Choi Y., Kwon S., Kim K., Kwon I.C. (2017). Dextran sulfate nanoparticles as a theranostic nanomedicine for rheumatoid arthritis. Biomaterials.

[91] Nguyen M.H., Lee S.E., Tran T.T., Bui C.B., Nguyen T.H.N., Vu N.B.D., Tran T.T., Nguyen T.H.P., Nguyen T.T., Hadinoto K. (2019). A simple strategy to enhance the in vivo wound-healing activity of curcumin in the form of self-assembled nanoparticle complex of curcumin and oligochitosan. Mater. Sci. Eng. C.

[92] Tietjen G.T., Bracaglia L.G., Saltzman W.M., Pober J.S. (2018). Focus on Fundamentals: Achieving Effective Nanoparticle Targeting. Trends Mol. Med..

[93] Martins J.C., do Amaral M.K.O., de Melo Amaral I.M., de Jesus Oliveira A.C., Leao A.D., Chaves L.L., de La Roca Soares M.F., Soares-Sobrinho J.L. (2026). Potential of polysaccharides as active targeting agents for nanoparticles toward biological receptors: A review. Int. J. Biol. Macromol..

[94] Yang J., Xiong K., Li T., Zhang M., Li Z., Wen Z., Jiang Y. (2025). Anti-inflammatory effects of natural polysaccharides: Molecular mechanisms and nanotherapeutic applications. Front. Immunol..

[95] Hu Q., Zhang F., Wei Y., Liu J., Nie Y., Xie J., Yang L., Luo R., Shen B., Wang Y. (2023). Drug-Embedded Nanovesicles Assembled from Peptide-Decorated Hyaluronic Acid for Rheumatoid Arthritis Synergistic Therapy. Biomacromolecules.

[96] Ma D.J., Li T.H., Yang S.Y., Yu J.J., Li S.T., Yu Y., Liu Y., Zang J., Kong L., Li X.T. (2025). Self-assembling Bletilla polysaccharide nanogels facilitate healing of acute and infected wounds via inflammation control and antibacterial activity. Int. J. Biol. Macromol..

[97] Zhang Q., Deng H., Luo R., Qi H., Lei Y., Yang L., Pang H., Fu C., Liu F. (2024). Oral food-derived whey protein isolate-Tremella fuciformis polysaccharides pickering emulsions with adhesive ability to delivery magnolol for targeted treatment of ulcerative colitis. Int. J. Biol. Macromol..

[98] Nabizadeh Z., Nasrollahzadeh M., Heidari F., Nasrabadi D. (2024). A drug-loaded nano chitosan/hyaluronic acid hydrogel system as a cartilage tissue engineering scaffold for drug delivery. Int. J. Biol. Macromol..

[99] Taha M.S., Padmakumar S., Singh A., Amiji M.M. (2020). Critical quality attributes in the development of therapeutic nanomedicines toward clinical translation. Drug Deliv. Transl. Res..

[100] Bastogne T. (2017). Quality-by-design of nanopharmaceuticals–A state of the art. Nanomedicine.

[101] Clogston J.D., Foss W., Harris D., Oberoi H., Pan J., Pu E., Torrico Guzmán E.A., Walter K., Brown S., Lim Soo P. (2024). Current state of nanomedicine drug products: An industry perspective. J. Pharm. Sci..

[102] Bernal-Chávez S.A., Del Prado-Audelo M.L., Caballero-Florán I.H., Giraldo-Gomez D.M., Figueroa-Gonzalez G., Reyes-Hernandez O.D., González-Del Carmen M., González-Torres M., Cortés H., Leyva-Gómez G. (2021). Insights into Terminal Sterilization Processes of Nanoparticles for Biomedical Applications. Molecules.

[103] Trenkenschuh E., Friess W. (2021). Freeze-drying of nanoparticles: How to overcome colloidal instability by formulation and process optimization. Eur. J. Pharm. Biopharm..

[104] Eknure C., Joshi V., Bachhav Y. (2026). Admixture Compatibility Studies in Parenteral Formulations: Packaging, Device, and Regulatory Perspectives. AAPS PharmSciTech.

